# ω-Tbo-IT1–New Inhibitor of Insect Calcium Channels Isolated from Spider Venom

**DOI:** 10.1038/srep17232

**Published:** 2015-11-27

**Authors:** Alexander N. Mikov, Irina M. Fedorova, Natalia N. Potapieva, Ekaterina E. Maleeva, Yaroslav A. Andreev, Alexey V. Zaitsev, Kira K. Kim, Eduard V. Bocharov, Timur N. Bozin, Dmitry A. Altukhov, Alexey V. Lipkin, Sergey A. Kozlov, Denis B. Tikhonov, Eugene V. Grishin

**Affiliations:** 1 Department of Molecular Neurobiology, Shemyakin–Ovchinnikov Institute of Bioorganic Chemistry RAS, Russia; 2 Laboratory of Biophysics of Synaptic Processes, I.M. Sechenov Institute of Evolutionary Physiology and Biochemistry RAS, Russia; 3 Department of Structural Biology, Shemyakin–Ovchinnikov Institute of Bioorganic Chemistry RAS, Russia; 4 NBIC Centre, NRC “Kurchatov Institute”, Russia

## Abstract

Novel disulfide-containing polypeptide toxin was discovered in the venom of the *Tibellus oblongus* spider. We report on isolation, spatial structure determination and electrophysiological characterization of this 41-residue toxin, called ω-Tbo-IT1. It has an insect-toxic effect with LD_50_ 19 μg/g in experiments on house fly *Musca domestica* larvae and with LD_50_ 20 μg/g on juvenile *Gromphadorhina portentosa* cockroaches. Electrophysiological experiments revealed a reversible inhibition of evoked excitatory postsynaptic currents in blow fly *Calliphora vicina* neuromuscular junctions, while parameters of spontaneous ones were not affected. The inhibition was concentration dependent, with IC_50_ value 40 ± 10 nM and Hill coefficient 3.4 ± 0.3. The toxin did not affect frog neuromuscular junctions or glutamatergic and GABAergic transmission in rat brains. Ca^2+^ currents in *Calliphora vicina* muscle were not inhibited, whereas in *Periplaneta americana* cockroach neurons at least one type of voltage gated Ca^2+^ current was inhibited by ω-Tbo-IT1. Thus, the toxin apparently acts as an inhibitor of presynaptic insect Ca^2+^ channels. Spatial structure analysis of the recombinant ω-Tbo-IT1 by NMR spectroscopy in aqueous solution revealed that the toxin comprises the conventional ICK fold containing an extended β-hairpin loop and short β-hairpin loop which are capable of making “scissors-like mutual motions”.

For many years, natural venoms have been valuable sources of biologically active compounds. The development of different analysis methods made it possible to unravel the complexity of spider venoms^1^,^2^,^3^,^4^,^5^,^6^ and identify action modes of isolated compounds on various molecular targets^7^,^8^,^9^,^10^,^11^,^12^,^13^,^14^. These investigations have shown that, among other venomous creatures, spiders produce the largest variety of venom compounds^15^. Taking into account the fact that spiders are the most abundant terrestrial predators^16^, this huge library of bioactive compounds remains poorly investigated. According to ArachnoServer (http://www.arachnoserver.org), the number of individual spider toxins investigated to date is 1403 from 97 different spider species^17^. Today, the average number of polypeptide toxins in spider venom is estimated at about 170 per species^18^. And according to The World Spider Catalog Version 16.5 (http://www.wsc.nmbe.ch/), there are currently 45,670 species of spiders on our planet (belonging to 114 different families); therefore, the estimated number of individual spider toxins seems to be quite amazing. Consequently, scientists have described only a tiny part of all spider venom toxins to date.

Regarding long and delicate tuning of venom composition by evolution, the most predominant spider toxins are those serving two main aims: predation of prey and protection of the spider itself^15^. Because insects are the main source of spider nutrition, a lot of insect-acting toxins have been detected by spider venom studies. Many of these toxins are insect specific and can, at low concentrations, cause paralysis or death of insects, but are not toxic to mammals^10^,^13^,^19^,^20^,^21^. The vast majority of insect-specific toxins are disulfide-stabilized polypeptides less than 10 kDa that shared one common fold named inhibitor cystine knot (ICK). Such polypeptide toxins act generally, whether on insect sodium, on insect calcium ion channels and rarer on potassium ones^10^,^11^,^13^. The action selectivity may be explained by structural differences of insect ion channels and their mammalian forms. Therefore, spider venom polypeptides may be regarded as a candidate base for the development of new insecticides^16^,^22^.

Development of such compounds is still very important because insects are acting in a disturbing way to different human activities. An increasing human population could require a large amount of food in the future. In this light, large annual crop yield reductions by insects pose a threat^16^. Moreover, such infectious diseases as malaria are transmitted by mosquitoes. In spite of some recent success in combating malaria^23^,^24^, there still remain many obstacles; therefore, mosquito control is a very urgent issue. A critical factor is also extending pest resistance to existing insecticides.

*Tibellus oblongus* (Walckenaer, 1802) belongs to the Philodromidae family and is distributed throughout the Holarctic^25^. *T. oblongus*, as another species of the Tibellus genus, is an active hunter that does not spin webs but actively pursues its prey. Papers describing any venom polypeptides for this spider have not been published to date, but cytochrome oxidase subunit 1, together with its gene and some ribosomal RNA genes, are published^26^. In preliminary experiments, we have observed strong insect toxicity for crude *T. oblongus* venom. And now we report on isolation, structure determination and recombinant production as well as electrophysiological characterization of a novel insecticidal polypeptide–ω-Tbo-IT1, which inhibits insect calcium channels, presumably the Ca_v_2 subtype.

## Materials and Methods

### Venom collection

The crude *T. oblongus* venom was purchased from Fauna Laboratories, Ltd. (Almaty, Republic of Kazakhstan). Female spiders were collected in the nearby Almaty region, and crude venom for an investigation was obtained by electrostimulation of several species.

All experiments were approved by the Animal Care and Use Committees of the Shemyakin-Ovchinnikov Institute of the Russian Academy of Sciences and Sechenov Institute of Evolutionary Physiology and Biochemistry of the Russian Academy of Sciences. Experiments were carried out in accordance with the guidelines approved by national Animal Protection Law, which is fully compatible with European Community Council directives 86/609/EEC.

All solvents, salts and reagents were purchased in the best quality available from certified suppliers. All gels and buffer solutions were prepared according to manufacturers’ manuals.

### Purification

ω-Tbo-IT1 toxin was fractionated from *T. oblongus* venom using a size-exclusion chromatography (SEC) and several steps of reverse-phase high pressure liquid chromatography (RP-HPLC). Soluble venom (2.7 mg in 5 ml) was applied to a Beckman TSK 2000SW column (7.5 × 600 mm) which was equilibrated with 20 mM sodium phosphate buffer (150 mM NaCl, pH 4.5). Separation was done at a flow rate of 0.5 ml/min, and chromatographic results were monitored at 214 nm. The active fraction was then applied on a Jupiter C_5_ (Phenomenex, USA) RP-HPLC column (4.6 × 250 mm) for separation in 0.1% v/v trifluoroacetic acid (TFA) buffer system in a linear gradient of acetonitrile concentration at a flow rate of 1 ml/min. The absorbance was monitored both at 210 nm and 280 nm. One active rough fraction was obtained as a result of the toxicity test on insects as described below. This active fraction was further fractionated on a Synergy Polar-RP400 (Phenomenex, USA) column (4.6 × 250 mm) in a linear gradient of acetonitrile (0.1% v/v TFA containing buffers) at a flow rate of 0.5 ml/min. Pure polypeptide elution time was monitored at the same wavelengths, 210 nm and 280 nm.

### Characterization of polypeptide structure

Dry polypeptide was dissolved in 40 μL of solution which contained 6M guanidine hydrochloride, 3 mM EDTA and 0.5 M Tris-HCl (pH 8.5). After that, a hundred-fold molar excess of dithiothreitol (DTT) was added, and reduction was performed at 40 °C in a nitrogen atmosphere for 4 hours. The polypeptide was alkylated by an isopropanol solution of 4-vinylpyridine (4 μL, 50% v/v; tenfold molar excess to DTT; 20 min in darkness) and purified from the reaction mixture on an RP-HPLC column. Proteolytic digestion of modified polypeptide was made by trypsin according to the procedure described^27^. Modified polypeptide molecules’ *N*-terminal sequence and tryptic peptide sequences were determined by Edman automated stepwise degradation using a Procise Model 492 protein sequencer (Applied Biosystems, USA) according to the manufacturer’s protocol.

### Mass spectrometry

Fractions after separation, pure and modified polypeptide, were analyzed by matrix-assisted laser desorption/ionization time-of-flight mass spectrometry (MALDI TOF-MS) on MALDI LR (Micromass UK) and Ultraflex TOF-TOF (Bruker Daltonik, Germany) instruments. ProteoMass peptide MALDI-MS calibration kit (with the range of 700–3500 Da) and protein MALDI-MS calibration kit (mass range of 700–66,000 Da; both by Sigma) were utilized for calibration of these spectrometers. Samples were prepared by the dried droplet method with *α*-cyano-4-hydroxycinnamic acid (in 50% acetonitrile +0.1% v/v TFA) matrix. Detection of molecular ions was performed in a linear positive mode.

### NMR spectroscopy and spatial structure calculation

1. Two-dimensional ^1^H-^1^H double quantum filtered correlation spectroscopy (DQF-COSY), total correlation spectroscopy (TOCSY; 80-ms mixing time), nuclear Overhauser effect spectroscopy (NOESY; 150-ms mixing time) and ^1^H-^13^C heteronuclear single quantum coherence spectroscopy (HSQC; at natural abundance) NMR spectra^28^ (Supplementary Figs S2, S3, S4, and S5) of 1.5 mM recombinant ω-Tbo-IT1 toxin (solubilized in H_2_O or D_2_O with 20 mM phosphate buffer, pH 5.8) were acquired at 12 °C and 20 °C on NMR spectrometer Varian NMR System 700 MHz (Agilent, USA; provided by NBICS-Center at NRC “Kurchatov Institute”). The Watergate^29^ technique was used to suppress strong solvent resonance. ^1^H and ^13^C chemical shifts were referenced relative to the trimethylsilyl propionate. ^1^H and ^13^C resonance assignments for ω-Tbo-IT1 were obtained by a standard procedure^30^ using NMR spectra in the CARA software^31^. The ^3^J_HNHα_ coupling constants were determined from a line shape analysis of the NOESY cross-peaks. The ^3^J_HαHβ_ coupling constants were measured using ACME program^32^ in the DQF-COSY spectrum acquired in D_2_O. Temperature coefficients of amide protons (Δδ^1^HN/ΔT) were measured in a temperature range from 12 °C to 20 °C using 2D TOCSY spectra. The slowly exchanging amide protons were identified at 12 °C by reconstituting of lyophilized ω-Tbo-IT1 in D_2_O and immediately recording the one-dimensional proton and two-dimensional ^1^H-^1^H TOCSY spectra.
2. The spatial structure calculation of ω-Tbo-IT1 (two variants: recombinant with homoserine lactone or native with methionine on *C*-terminus) was performed using the simulated annealing/molecular dynamics protocol as implemented in the CYANA software package^33^. Upper inter-proton distance constraints were derived from nuclear Overhauser effect (NOE) cross-peaks *via* standard CYANA calibration procedure. Torsion angle restraints and stereospecific assignments were obtained by the analysis of local conformation in CYANA using sequential NOE data and J coupling constants. All Xxx-Pro peptide bonds were clearly identified as *trans* on the basis of characteristic NOE contacts^30^. The disulfide binding pattern (Cys residues 1–21, 8–25, 20–40, and 27–38) was uniquely determined from preliminary structure calculations, and corresponding distance restraints were introduced in CYANA software. Other possible disulfide bond connectivity patterns were tested in a course of spatial structure calculations, but they did not correspond to the experimental NMR data. The 22 amide protons having temperature gradients less than 5 ppb/K and/or slow exchange with water protons were supposed to participate in hydrogen bonding and assigned as donors of backbone hydrogen bonds with related hydrogen-acceptor partners on the basis of preliminary structure calculations. Corresponding hydrogen bond restraints were employed in subsequent calculations for *d*(O,N), and *d*(O,H^N^) distances in accordance with hydrogen bond distance criteria in CYANA software. NOESY spectra back calculation was used to verify that the calculated structure did not have proton-proton contacts that were not seen in the spectra. At the final stages of structure calculations, inter-proton lower distance restraints of 4 Å were employed for such contacts. In the final calculation stage, the standard CYANA simulated annealing protocol was applied to 100 random structures using angle and distance restraints, and resulting 20 NMR structures of ω-Tbo-IT1 with the lowest target functions were selected.

Visual analysis of calculated structures and figure drawings were performed using the MOLMOL software^34^.

### Precursor determination

Total RNA was purified from the venom glands of *T. oblongus* using Trisol® Reagent (Ambion, Canada) according to manufacturer protocol. Total cDNA was synthesized from 5 μg of total RNA, using the MINT kit (Evrogen, Russia) following the manufacturer recommendations. Rapid amplification of cDNA ends was carried out using the universal primer T7cap (GTA ATA CGA CTC ACT ATA GGG CAA GCA GTG GTA ACA ACG CAG AGT) and degenerated primers To1 (TGT GCC AGC AAG AAY GAR MGN TGY GGN AAY) and To2 (GCG AGC AAG AAT GAR MGN TGY GGN AAY GCN) for 3′-terminus determination (3′-RACE) and To3 (ACG CGC AGT TTC TTR CTY KCN ACR CCN) for 5′-terminus determination (5′-RACE). DNA sequencing was carried out on ABI PRISM 3100-Avant.

### Gene construction

Production of a recombinant analogue of the toxin proceeded utilizing the pET-32-b(+) vector (Merck Biosciences, USA). Toxin gene was synthesized by PCR reaction in two stages on the basis of overlapping primers having an additional Met-codon for BrCN cleavage and a stop codon. The following primers were employed: TboIT1-D0 (AGC CAT GGC GAT GTG CGC TTC TAA GAA CGA ACG TTG), TboIT1-D1 (TCT AGG AAC GAA CGT TGC GGT AAC GCT CTG TAC GGT ACC AAA GGT CCG), TboIT1-D2 (GGT AAA TGC ATC TGC CGT ACC GTT CCG CGT AAA GGT GTT AAC TCT TGC), TboIT1-R4 (ACG GCA GAT GCA TTT ACC GTT GCA GCA ACC CGG ACC TTT GGT ACC GTA C), and TboIT1-R5 (CTC TCG AGT CAC ATG CAA CGG CAA GAG TTA ACA CC). PCR was performed using a (5:1) mixture of Taq DNA polymerase (Evrogene, Russia) and Pfu DNA polymerase (Promega, USA) under the following conditions: incubation at 94 °C for 5 min; 4 cycles (first stage PCR) or 24 cycles (second stage PCR) with each cycle including denaturation at 94 °C for 20 sec, annealing at 56 °C for 20 sec and extension at 72 °C for 20 sec; an additional extension step at 72 °C for 5 min and cooling at 10 °C for 1 min. Amplified products were separated in a 1% agarose gel, and a target product was obtained by a GeneJet™ gel extraction kit (Thermo Scientific Fermentas, USA). The resulting gene product was ligated to the pET-32-b(+) vector on the EcoRV restriction site by blunt ends. Sequences of the genes were confirmed after cloning in XL21 *E. coli* cells.

### Recombinant analog production

*E. coli* BL21(DE3) cells were transformed with expression vectors by electroporation. Transformed cells were cultured in LB medium with ampicillin (100 mg/ml) at 37 °C up to solution density 0.6–0.7 AU at 600 nm. T7-promoted expression was induced by addition of isopropyl-1-thio-β-D-galactopyranoside (IPTG) up to 0.2 mM concentration. Expression was performed for 12 h at 24 °C, after which cells were harvested and re-suspended in chromatogram buffer (300 mM NaCl, 20 mM Tris-HCl pH 7.5). The suspension was ultrasonicated, and the supernatant, after centrifugation (15 min at 14,000*g*), was applied to TALON Metal Affinity resin (Clontech Laboratories, USA). Fusion protein purification was made in accordance with the manufacturer’s manual. Protein cleavage was performed overnight at room temperature in the dark in accordance with method^35^. HCl to a final concentration of 0.2 M and CNBr with a molar ratio to a fusion protein of 600:1 were added. Final purification of the recombinant polypeptide was made by RP-HPLC on Jupiter C_5_ (Phenomenex, USA) column, and then its identity to natural polypeptide was confirmed by MALDI-MS.

### Insect-toxicity tests

Toxicity was measured on *M. domestica* larvae of about 50 mg by weight. Lyophilized samples were dissolved in pure water, and fixed aliquots were injected into larvae bodies using a 10 μL syringe (Hamilton, USA). To estimate concentration dependent effect, doses of 0.375, 0.75, 1.25, 3.0, 5.0, 7.0, 9.0, 12, 20, 25, 30, 40 and 60 μg/g were used. The paralyzing effect development was monitored during a period of 48 h. Each dose was measured on a group consisting of 16–20 larvae. The test was repeated five times, and standard errors of the mean were calculated. Toxicity was also measured on *G. portentosa* cockroaches by the same protocol using 1.25, 2.5, 5.0, 10, 20, 40, 80 μg/g dose equivalents. In this case, the paralyzing effects development was monitored during a period of 24 h.

### Electrophysiology

The effects on insect neuromuscular transmission were studied on late third stage larvae of *Calliphora vicina*. After dissection, the internal organs were removed so that the preparation consisted only of muscles attached to the cuticle. The ventral ganglion was excised, and the segmental nerves were stimulated through a suction electrode. Recordings were made from ventral longitudinal fibers. To eliminate electrical contacts of the recorded fiber with its neighbors, the latter were dissected. The preparation was perfused with a saline solution containing (in mM): 172 NaCl, 2.5 KCl, 0.5 CaCl_2_, 8 MgCl_2_, 2.4 NaHCO_3_, 0.3 H_2_PO_4_, and 52 sucrose. pH was adjusted to 7.2 with NaOH or HCl. Experiments were performed at room temperature (20–24 °C). The excitatory postsynaptic currents (eEPSC) were evoked by nerve stimulation (0.5 Hz). Voltage-activated currents in muscle cells were evoked by 500 ms depolarizing voltage pulses from holding potential −90 mV to 0 mV. Potassium currents were inhibited both by 2 mM 4-aminopyridine (4-AP) and 10 mM tetraethylammonium. Sodium currents were eliminated by 10 mM lidocaine. To prevent muscle contraction, external Ca^2+^ was replaced by 10 mM Ba^2+^.

Adult male cockroaches of species *Periplaneta americana* were taken from stock colonies maintained at 29 °C with a photoperiod of 12 h light and 12 h darkness. Animals were cold anesthetized and immobilized on a dissection dish dorsal side up. Legs, dorsal cuticle gut and dorsolongitudinal muscles were removed to isolate the nerve cord. The preparation was placed in an experimental chamber with a solution containing (in mM): 160 *N*-methyl-D-aspartic acid (NMDG), 5 CaCl_2_, 10 HEPES, 20 TEA and 14 4-AP, with pH 7.3. Large (>50 μM) neurons were isolated by vibrodissociation from the terminal abdominal ganglion as described^36^ that use a local application of mechanical vibration directly on a ganglion instead of a tissue enzymatic treatment. The whole-cell voltage clamp recordings of Ca^2+^-currents were done with the pipette solution (in mM): 130 cesium methanesulfonate (CsMeSO_4_), 8 NaCl, 10 HEPES, 4 MgATP, 0.3 NaGTP, 0.5 ethylene glycol tetraacetic acid (EGTA) and 6 *N*-(2,6 dimethylphenylcarbamoylmethyl)-triethyl-ammonium bromide (QX314), with pH 7.3, using EPC8 amplifier (HEKA Elektronik). Currents were evoked by 500 ms depolarizing voltage pulses from holding potential −90 mV to 0 mV.

A cholinergic transmission was studied on a *musculus sartorius* neuromuscular preparation of the common frog *Rana temporaria*. The preparations were superfused with solution (in mM): 113 NaCl, 2.5 KCl, 0.6 CaCl_2_, 3 NaHCO_3_ and 4 MgCl_2_ (pH 7.3). Currents were recorded by a conventional two-electrode voltage clamp using an Axoclamp 2B (Axon Instr., USA) amplifier. The data were filtered at 2 kHz and stored on a computer.

Effects on (gamma-aminobutyric acid-ergic) GABAergic and glutamatergic transmission were studied on brain slices of male *Wistar* rats (20–22 postnatal days). Animals were killed by cervical dislocation and then were decapitated. Slices containing medial frontal (prelimbic) cortex were cut with a vibratome (Vibroslice 752M, Campden Instruments, UK) and incubated at room temperature for at least 1 h and then were transferred to a recording chamber perfused with artificial cerebrospinal fluid (ACSF) at 24–25 °C. Whole-cell recordings were made from layer 3 pyramidal neurons which were identified visually using infrared transmitted illumination from Axioscop microscope (Karl–Zeiss, Germany) equipped with differential interference contrast optics and Sanyo video camera (model VCB-3512P, Japan) for contrast enhancement. Pyramidal neurons were clamped at −80 mV during recording, and synaptic responses were evoked with a monopolar glass electrode. For recording of eEPSC in rat brain neurons, the pipette solution contained (in mM): 114 K-gluconate, 6 KCl, 10 HEPES, 4 ATP-Mg and 0.3 GTP (pH was adjusted to 7.25 with KOH). To reverse and increase the amplitude of GABA_A_ receptor-mediated evoked inhibitory post synaptic currents (eIPSCs) in postsynaptic cells at negative membrane potential, we used an internal solution of high chloride concentration, which contained (in mM): 120 KCl, 10 HEPES, 4 ATP-Mg and 0.3 GTP (pH was adjusted to 7.25 with KOH). The external solution contained (in mM) 143 NaCl, 5 KCl, 2.5 CaCl_2_, 2 MgCl_2_, 10 D-glucose, 10 HEPES (pH was adjusted to 7.4 with HCl). All recordings were made on an EPC8 amplifier (HEKA Elektronik, Germany) operated in a bridge-balance mode with capacitance neutralization. Signals were filtered with 5 kHz and digitized with a sampling frequency of 10 kHz for analysis.

### Computation

Homological polypeptides search in Uniprot Protein Knowledgebase was done using BLASTp tool (BLOSUM-80 matrix). Offline computation, sequence alignments and *E. coli* expression codon optimization were performed with Lasergene® (DNAStar, USA) programs.

## Results

### Toxin isolation

Crude *T. oblongus* venom was selected as a source of insect-selective components because it had shown high toxicity to insects in a preliminary test (data not shown). For isolation of active compounds, we employed the proven tactic based on step-by-step separation by different chromatographic methods following biological activity trials of fractions^21^,^37^. Acute toxicity to insects was used as the test.

**Figure 1:**
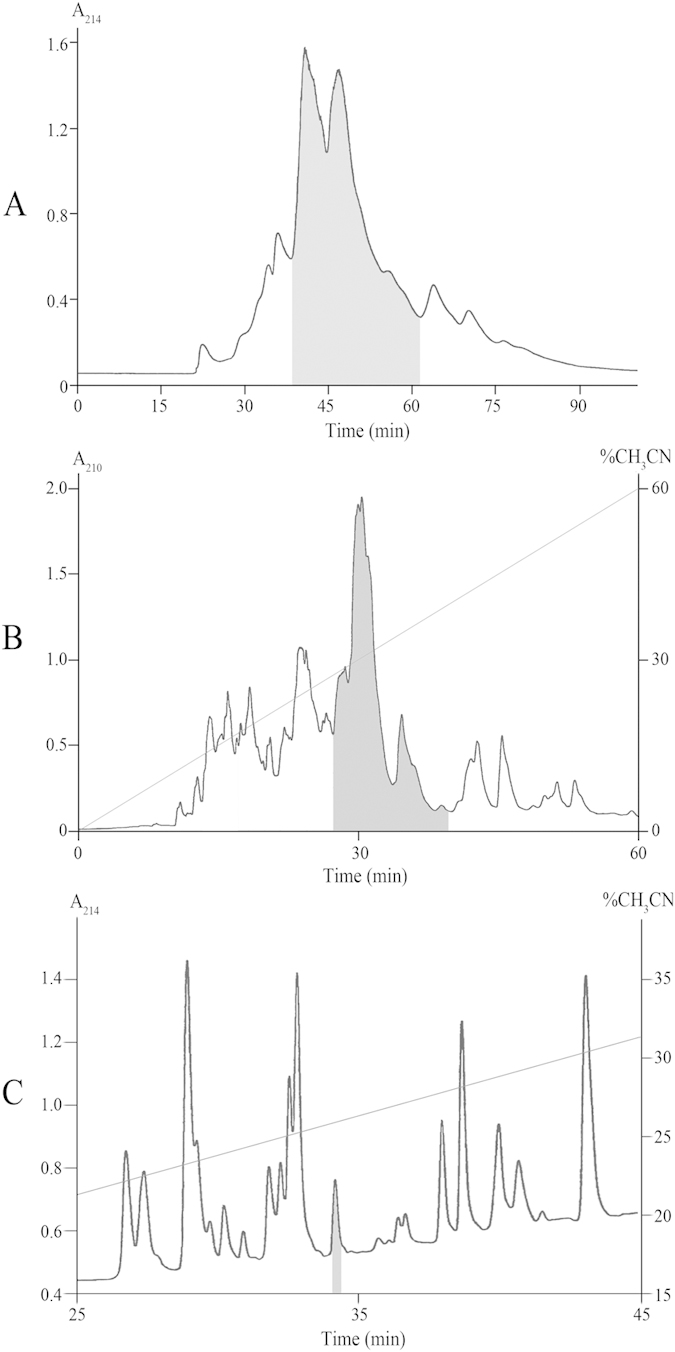
Multistage isolation of ω-Tbo-IT1 from crude venom by chromatoraphy. (**A**) On a size-exclusion column TSK 2000SW column (Beckman, 7.5 × 600 mm) in 20 mM sodium phosphate (pH 4.5) with 150 mM NaCl buffer, at flow rate 0.5 ml/min; (**B**) on RP-HPLC column Jupiter C_5_ (Phenomenex, 4.6 × 250 mm) in 0.1% TFA containing buffers with a linear gradient of acetonitrile concentration at the flow rate 1 ml/min; (**C**) on Synergy Polar-RP400 column (Phenomenex, 4.6 × 250 mm) in 0.1% TFA containing buffers with a linear gradient of acetonitrile concentration at the flow rate 0.5 ml/min. Elution time of fractions toxic to insects are shadowed.

Measured activity was concentrated in polypeptide compounds that were separated from low and high molecular weight components by SEC (Fig. 1A). Rough separation by RP-HPLC (Fig. 1B) resulted in a selection of a moderately hydrophobic fraction that finally was separated into individual polypeptides (Fig. 1C). The purity of the most active polypeptide (ω-Tbo-IT1) obtained after third separation was confirmed by MALDI-MS that detected one component with molecular weight 4332.8 Da.

### Primary structure determination

*N*-terminal Edman sequencing of the isolated polypeptide revealed first eleven residues to be: CASKNERCGNA. The mass difference between native and alkylated polypeptides prove an eight-cysteine content that is very common among spider toxins having ICK folding^38^,^39^. To complete structure elucidation, rapid cDNA ends amplification (RACE) with following sequencing approach was utilized.

**Figure 2:**
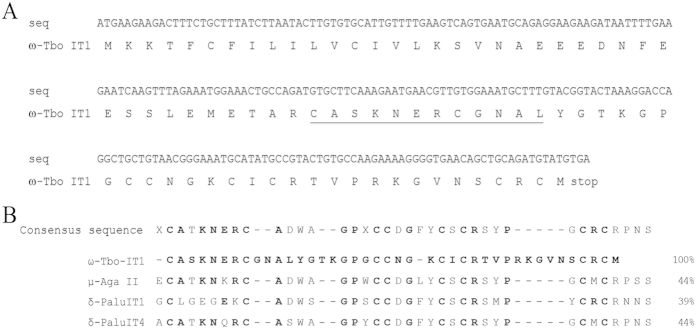
Primary structure features of the novel polypeptide toxin: (**A**) cDNA coded precursor protein sequence and derived protein structure of ω-Tbo-IT1, where residues equal to those defined by Edman degradation are underlined. (**B**) Alignment of mature ω-Tbo-IT1 sequence with three best BLASTp hits retrieved from UniProt databank. Residues similar to ω-Tbo-IT1 are shown by bold letters.

As a result, it was deduced that ω-Tbo-IT1 precursor protein consisted of the signal peptide, the propeptide fragment and 41 residue-length mature sequence followed by a stop codon (Fig. 2A). The prepropeptide structure was in good accordance with spider toxin maturation principles^40^,^41^, and no other post-translation modifications were identified. Molecular weight difference for native toxin compared with deduced sequence was 0.3 Da, and tryptic peptide masses were also in close agreement.

The BLASTp search for primary structure homologs for mature polypeptide sequence revealed some structural identity with three spider toxins (Fig. 2B). There was one insect sodium channel inhibitor μ-agatoxin II from funnel-web spider *Agelenopsis aperta* venom^42^, and there were two inhibitors of insect sodium channels inactivation, δ-palutoxin IT1 and δ-palutoxin IT4 from *Pireneitega luctuosa* spider venom^43^.

### Recombinant analogues production

To produce a sufficient amount of ω-Tbo-IT1 for further analysis, a recombinant analog was synthesized in *E. coli* BL21(DE3) cells. Thioredoxin was chosen as a fusion partner for expression, as it had shown a good potency for enhancing polypeptide toxin expression earlier^44^,^45^,^46^. Thioredoxin–ω-Tbo-IT1 fusion protein production and purification were followed by CNBr cleavage to release target polypeptide finally purified by RP-HPLC. The yield of the purified recombinant polypeptide was very poor –0.46 mg/L of cell culture. The molecular weight of the recombinant product was equal to the natural one but with a difference referred to homoserine lactone on the *C*-terminus that is a common modification for CNBr treated polypeptides. The identity of recombinant analog by biological function to the natural ω-Tbo-IT1 was confirmed by biological tests both in acute toxicity on *M. domestica* larvae and in electrophysiological assays on *C. vicina* preparations.

### Toxicity assay

Evaluative toxicity tests were performed for identification of the most toxic fractions during whole process of *T. oblongus* spider venom fractionation when small lyophilized part of each fractions was injected in order to give a rough estimate of its activity.

**Figure 3:**
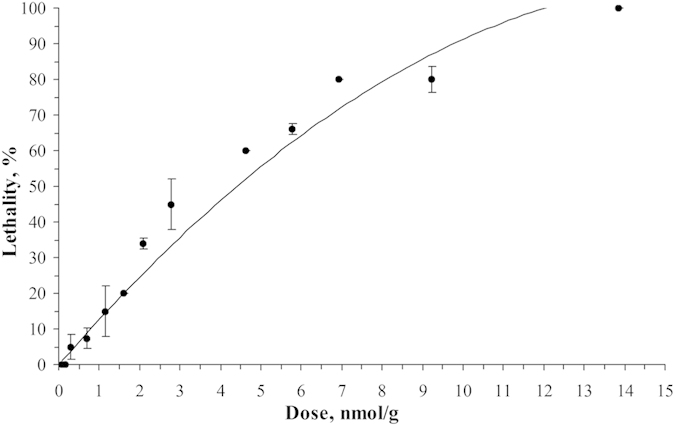
Dose-response curve resulting from injection of ω-Tbo-IT1 into *M. domestica* larvae (n = 5). Standard errors of the mean are shown.

Crude venom toxicity to *M. domestica* larvae was estimated as LD_50_ 10 μg/g for polypeptide fractions (separation steps one and two). Doses were a little bit higher for pure toxin ω-Tbo-IT1, which possessed toxic activity of about 15 μg/g (data not shown). More precise measurements were made for recombinant analogs (Fig. 3) that have demonstrated activity equal to those measured earlier for the native toxin. The LD_50_ dose calculated from this dose-dependent curve was approximately 950 ng/larvae, that is, equal to 19 μg/g or 4.4 nmol/g of body weight.

Crude *T. oblongus* venom injection displayed immediate paralysis approximately one minute after injection. After this time elapsed, larvae revealed brief physical activity of about thirty seconds in duration, followed by paralysis. This paralysis lasted for about 2 h, and then if they had been subjected to a low dose of venom, larvae started responding weakly to stimuli; but they died at higher doses. The same effect was observed for active fraction and the pure toxin as well as for recombinant analogue. For low doses of recombinant toxin (less than LD_50_), time for paralysis to occur increased from 30 s to 2 min.

Paralyzing action towards cockroaches was demonstrated both on adult and juvenile specimens of *G. portentosa*. Dose 20 μg/g resulted in fast partial paralysis of adult cockroaches, which died 24 h after injection. Lower doses did not induce any paralysis or lethal indications while doses 20, 40 and 80 μg/g led to 50, 75 and 100% mortality during 24 h. So the activity of the toxin to both analyzed insects was similar.

### Electrophysiology

**Figure 4:**
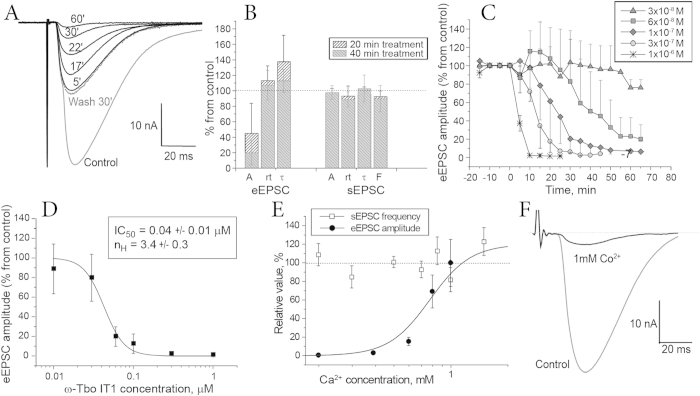
Action of ω-Tbo IT1 on *C. vicina* neuro-muscular junction. (**A**) Reversible time dependent inhibition of eEPSC (30 min washout presented) by 0.1 μM natural ω-Tbo-IT1. (**B**) Parameters chart for measured amplitude (**A**), rise time (rt), decay time constant (dt), and frequency of sEPSC (**F**) after 0.1 μM natural toxin application. Standard deviation are shown (n = 6). (**C**) Concentration dependent eEPSC amplitude decrease and (**D**) inhibition curve fitted by Hill equation evoked by the toxin application. Standard deviation are shown (n = 5). (**E**) Ca^2+^ concentration in external solution controls the eEPSC amplitude but not the sEPSC frequency (n = 6). (**F**) Co^2+^, the non-selective Ca^2+^ channel blocker, inhibits the eEPSC like ω-Tbo-IT1 (see panel **A**).

Activity of native toxin was manifested as reversible inhibition of eEPSC in *C. vicina* neuromuscular junction (Fig. 4A). The inhibition was not accompanied by significant change of rise time (rt) or decay (τ) of the eEPSC (Fig. 4B). Parameters of spontaneous EPSC (sEPSC) were not affected by the toxin, suggesting that the effect was due to the quantum content decrease. Interestingly, frequency of sEPSC was also unaffected. Thus, the toxin strictly discriminated evoked and spontaneous release. Recombinant analog effect on neuro-muscular junction was identical to the natural toxin (data not shown). Stock solution of recombinant polypeptide was used for the concentration dependence analysis that resulted in the calculation of IC_50_ value 40 ± 10 nM and Hill coefficient 3.4 ± 0.3 (Fig. 4C,D).

The revealed difference between the toxin effects on evoked and spontaneous neuromediator release suggests that presynaptic Ca^2+^ channels are the most obvious molecular target of the action. Indeed, for the classical blocker of these insect presynaptic calcium channels palutoxin-II (PLTX-II), similar effects were measured in an electrophysiological study on *Drosophila melanogaster* neuromuscular junctions^47^. To check the regulatory role of Ca^2+^ ions on the eEPSC amplitude and sEPSC frequency, we varied external Ca^2+^ concentration in our preparation. Decrease of Ca^2+^ concentration caused steep decrease of eEPSC amplitude with the apparent EC_50_ of 0.7 ± 0.2 mM and Hill coefficient 3.8 ± 0.2 (Fig. 4E). The Hill coefficient was similar to the one calculated for ω-Tbo-IT1 inhibition. On the contrary, the frequency of sEPSC was found insensitive to the Ca^2+^ concentration (Fig. 4E). To further confirm that toxin action mode may be due to inhibition presynaptic calcium channels, we studied the action of Co^2+^ ions, which is a non-selective Ca^2+^ channel blocker (Fig. 4F). 1 mM Co^2+^ caused strong inhibition of eEPSC amplitude with only minor action on the frequency and other characteristics of sEPSC. Thus, the effect of ω-Tbo-IT1, the effect of Ca^2+^ reduction and the effect of Co^2+^ are similar.

**Figure 5:**
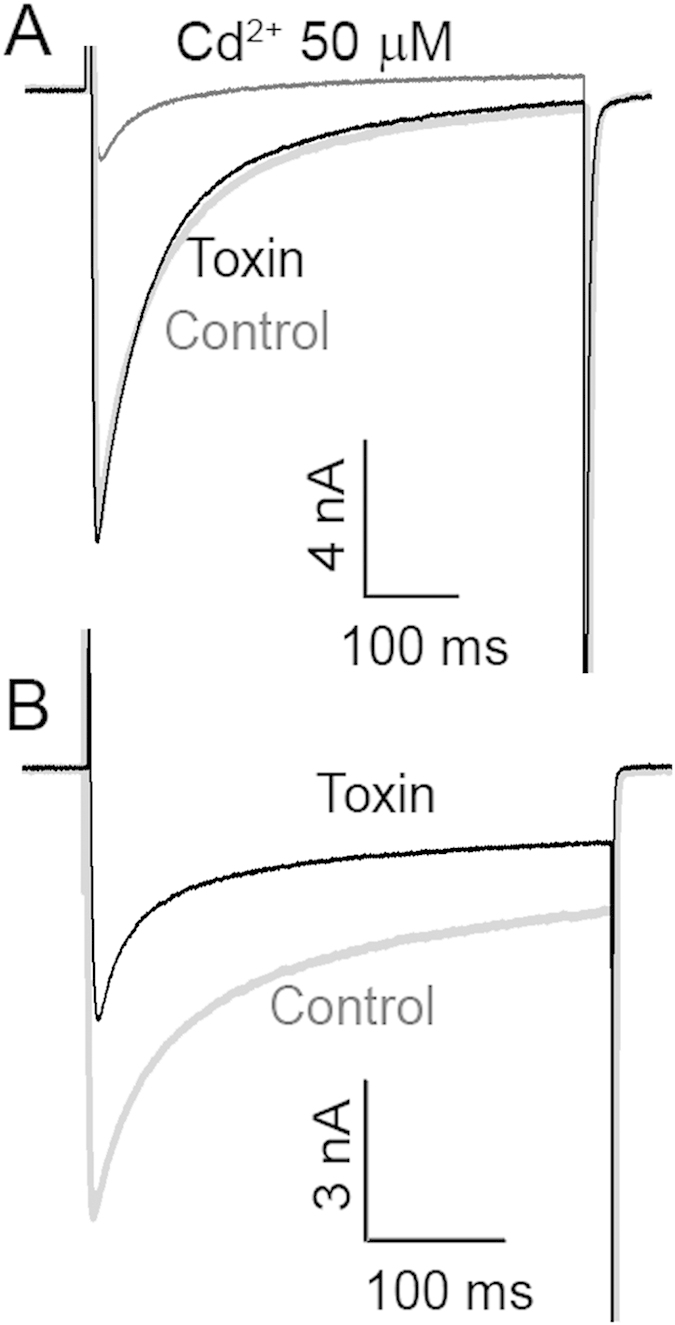
ω-Tbo IT1 action on Ca^2+^ channels in cockroach neurons. Representative recordings of Ca^2+^ currents evoked by 500 ms membrane depolarization from –90 mV to 0 mV in control (gray) and 1 μM concentration of ω-Tbo-IT1. (**A**) Example of the toxin-sensitive current. (**B**) Example of the toxin-insensitive current, which however is inhibited by Cd^2+^.

To directly demonstrate action of ω-Tbo-IT1 on presynaptic Ca^2+^ channels, we used neurons isolated from the abdominal ganglion of the cockroach *P. americana*. The Ca^2+^ currents were evoked in these neurons by depolarization from −90 to 0 mV. In all 28 neurons tested 50 μM Cd^2+^ caused strong and reversible current inhibition. In 16 cells the currents were completely insensitive to ω-Tbo-IT1 in 1 μM concentration (Fig. 5A). In the other cells Ca^2+^ currents demonstrated significant inhibition 48 ± 17%, (n = 12) (Fig. 5B). The inhibition was partially reversible during a 20 min period. Thus, among the different subtypes of Ca^2+^ channels expressed in cockroach neurons, there is a significant fraction, which is sensitive to ω-Tbo-IT1.

**Figure 6:**
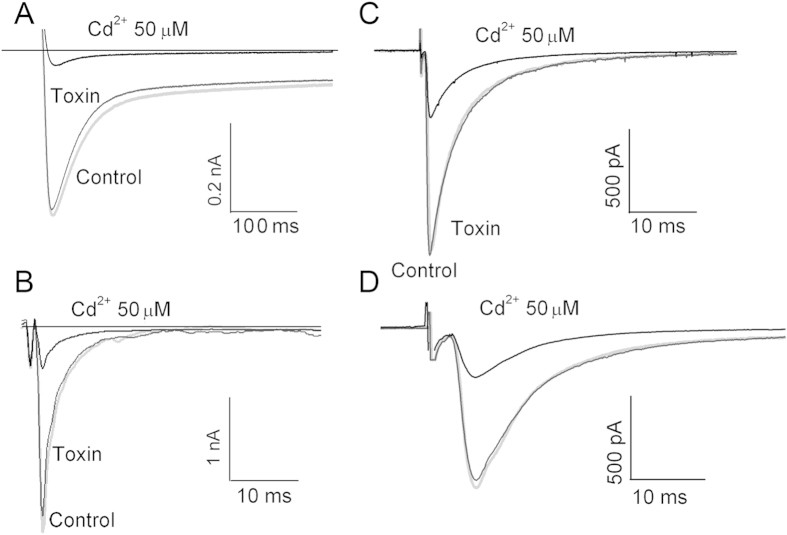
Selectivity of ω-Tbo-IT1 action. Two-electrode voltage clamp records in the fly muscle cell for the Ba^2+^ mediated currents evoked by voltage jumps from −90 mV to −20 mV (**A**) as well as synaptic currents in frog neuro-muscular junction (**B**) and both GABA-ergic (**C**), and glutamate-ergic transmission (**D**) in rat brain slices were not affected after application of ω-Tbo IT1 in 1 mM concentration. Currents inhibited by 50 mM Cd^2+^ and control currents (grey) are presented.

To analyze selectivity of ω-Tbo-IT1 and to reveal other possible targets, several additional preparations were used. In all preparations, a 1 μM concentration, which caused fast and complete inhibition of eEPSC on the fly neuromuscular synapse, was used. First, we studied ω-Tbo-IT1’s effect on Ca^2+^ channels in insect muscle. Fig. 6A demonstrates the lack of toxin effect on barium-mediated currents (n = 6), which were readily blocked by 50 μM Cd^2+^. Thus, a subtype of Ca^2+^ channels sensitive to ω-Tbo-IT1 has neuronal specificity.

To determine the toxin’s selectivity for insects, we tested ω-Tbo-IT1 action on non-insect preparations such as frog neuromuscular junction (Fig. 6B) and rat brain glutamate-mediated (Fig. 6C) and GABA-mediated (Fig. 6D) neuronal synaptic transmission in the prefrontal cortex. To isolate (α-amino-3-hydroxy-5-methyl-4-isoxazolepropionic acid) AMPA receptor-mediated component of EPSCs, all recordings were performed in the presence of GABA_A_ receptor antagonist bicuculline methyl-bromide (10 μM) and (isopropyl-1-thio-β-D-galactopyranoside-) NMDA-receptor antagonist *D*-(-)-2-amino-5-phosphono-pentanoic acid (d-AP5, 50 μM). To isolate GABA_A_, receptor-mediated inhibitory post-synaptic current recordings were performed in the presence of AMPA-receptor antagonist 6-cyano-7-nitroquinoxaline-2,3-dione (CNQX, 10 μM) and d-AP5 (50 μM). No significant action of the toxin was revealed in all these preparations (n = 6, for each preparation). In all cases 50 μM Cd^2+^ strongly inhibited the post-synaptic currents. Thus, we identified ω-Tbo-IT1 to be a specific inhibitor of insect presynaptic Ca^2+^ channels.

### Spatial structure of ω-Tbo-IT1

**Figure 7:**
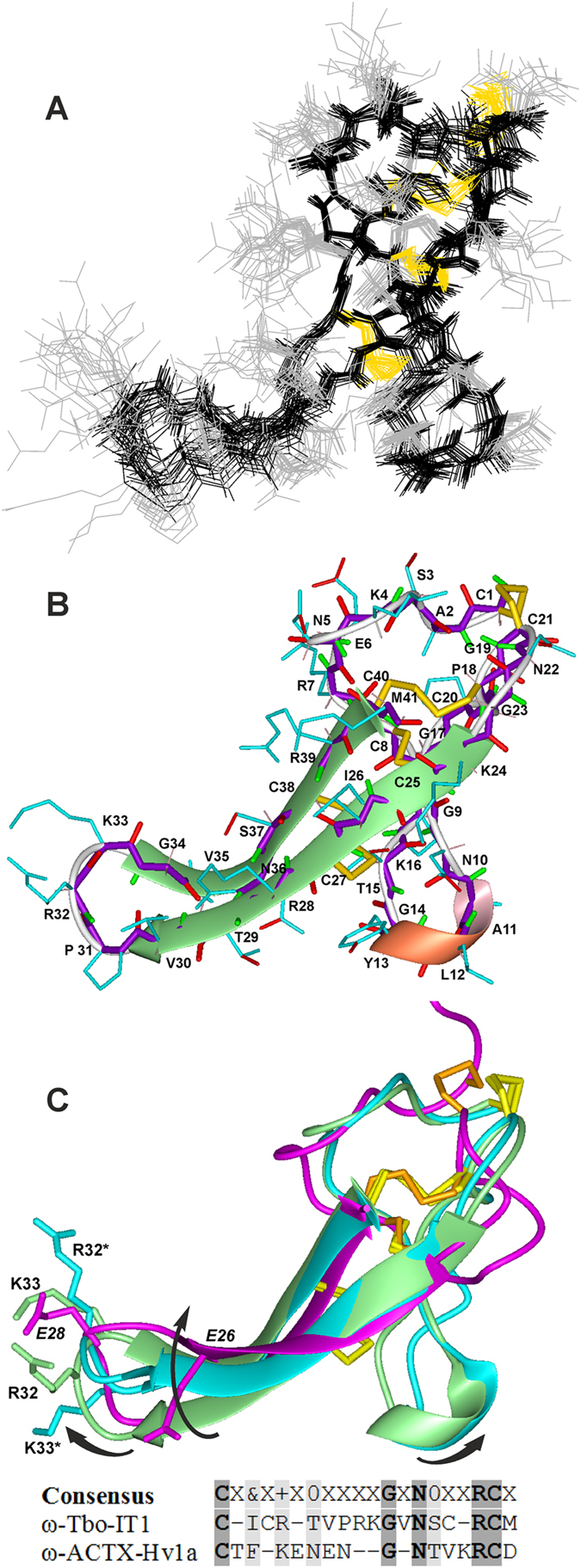
Spatial structure of ω-Tbo-IT1. (**A**) Ensemble of 20 NMR-derived structures of ω-Tbo-IT1 after superposition of the backbone atoms of the “cystine knot” residues (regions 1–8, 20–27 and 38–40). The heavy side chain and backbone bonds are shown in *black* and *gray*, respectively. (**B**) Ribbon representation of the ω-Tbo-IT1 structure. Extended β-sheet and 3_10_-helical turn of the major and small β-hairpin loops are highlighted in *green* and *pink*, respectively. The backbone and side-chain heavy bonds are shown in *violet* and *cyan*, respectively. The carbonyl, carboxyl, amide and disulfide bonds are additionally depicted in *red*, *green* and *yellow*, respectively. (**C**) Spatial structure alignment of the major (in *green*) and minor (in *cyan*) conformation of ω-Tbo-IT1 with ω-ACTX-Hv1a (in *magenta*). The arrows indicate the postulated twist and scissors-like mutual motions of the β-hairpin loops of ω-Tbo-IT1. The side-chains of the residues alternatively charged the tips of ω-Tbo-IT1 (positive R32/R32* and K33/K33* for major/minor conformations) and ω-ACTX-Hv1a (negative E26 and E28) are shown. In the *bottom*, sequence alignment for region of β-hairpin tip of two insect calcium channel inhibitors (ω-Tbo-IT1 and ω-ACTX-Hv1a) is presented. Positions of positively charged residues marked in the consensus as “+”, polar uncharged residues as “0” and hydrophobic–residues as “&”. Completely homologous residues are bold and shaded. Similar residues are only shaded.

Spatial structure of recombinant ω-Tbo-IT1 was studied by NMR spectroscopy in aqueous solution at pH 5.8. The set of 20 structures (Fig. 7A) was calculated using the following NMR experimental data: upper and lower NOE-based distance restraints, J coupling-based torsion angle restraints and hydrogen bond restraints (see a survey of the structural statistics in Table 1). The obtained structural ensemble (Fig. 7A) demonstrates no large distance violations, good Ramachandran statistics and quite low values of the root mean square deviation (RMSD) for backbone and heavy atoms (Table 1).

The ω-Tbo-IT1 structure (Fig. 7B) involves two loops protruding from a “globular core” cross-linked by three disulfide bonds (Cys1-Cys21, Cys8-Cys25 and Cys20-Cys40) presenting the “cystine knot” arrangement (I–IV, II–V, III–VIII) typical for spider venom toxins with eight cysteines. The toxin is therefore concluded to assume the conventional ICK fold. The short β-hairpin loop (Gly9-Lys16) has on its tip a reverse turn (Ala11-Gly14) in the 3_10_-helix conformation with the capping side chain to backbone (Asn10-Leu12) hydrogen bond. The major β-hairpin is formed by two extended β-strands (Lys24-Val30 and Gly34-Met41) having bulge regions (Thr29-Val30 and Gly34-Asn36) which are connected by an inverse γ-turn (Pro31-Lys33) on the hairpin tip and “stapled” by the additional disulfide bond (Cys27-Cys38) in the center. In addition to 4 disulfide bonds, the toxin structure is stabilized by 21 backbone-to-backbone hydrogen bonds.

Nevertheless, some heterogeneity exists in the toxin spatial structure, which is revealed by the disproportional peak doubling and resonance broadening observed in the NMR spectra (Supplementary Figs S1, S2, and S3) for some residues situated near the “cystine knot” disulfide bridges, the short β-hairpin loop and the bulge regions of the extended β-strands. Enhanced intramolecular mobility with slow (in the NMR time scale) conformational exchange between the major and minor similar forms can be caused by disulfide bridge isomerization or hydrogen bond switching. These would result in moderate mutual movements of the β-hairpin loops and alternative twisting of the β-sheet structure in the major β-hairpin loop, which are observed in the structural ensemble of ω-Tbo-IT1 (Fig. 7A,B). At the same time, the obtained structure set reveals also that the β-hairpin loops may be connected by the hydrogen bonding of the side chain of Asn36 with the backbone carbonyl group of Tyr13 and the side-chain hydroxyl group of Thr15 that would restrict the scissors-like mutual motions of the β-hairpin loops, in favor of the major conformation.

## Discussion

Initially, the present work was aimed at finding new insect-selective toxins with acute toxicity toward pests. *T. oblongus* venom was chosen as a source based on the hunting features of this spider. The 41-residue toxin called ω-Tbo-IT1 was isolated, and it demonstrated significant toxicity to insects and neutrality to vertebrates.

### Primary structure and toxicity

The toxin ω-Tbo-IT1 has a classical ICK motif and demonstrates moderate sequence homology with the insect sodium channel inhibitors from the venom of funnel-web spider *Agelenopsis aperta*^42^ and Australian tangled nest spider *Pireneitega luctuosa*^43^. Following structural classification for spider venom peptide^48^, this new insect toxin belongs to structural class 2.1.1.0, for members of which classical distribution of cysteines is characteristic. Using another proposed classification^49^, a novel toxin may be named ω-philotoxin-To1a, which reflects biological action on Ca^2+^ channels and appurtenance of source spider to Philodromidae family.

Toxin primary structure is substantially different compared to other spider calcium toxin structures. As shown in Fig. 2B, three polypeptides share homology of less than 50%, and the maximal disagreement is situated in the loop sequences located between Cys II-Cys III and Cys VI-Cys VII. Both regions are shorter in other ICK toxins under consideration; these regions were detected by NMR as regions of ω-Tbo-IT1 with intramolecular mobility (3_10_-helix and tip of β-hairpin loop on Fig. 7B,C). This allows making the assumption about the unique evolutionary advantage of ω-Tbo-IT1 structure over other spider ICK family members. It may mean that twist- and scissors-like motions help ω-Tbo-IT1 adjust its binding site to the environment, so the toxin likely has dynamic rather than static epitope.

As noted above, the primary structure homology is not related to the biological properties of toxins. However, it was interesting to compare the acute toxicity measured in insects to the nearest structural homologs and for some of the most active insect sodium channel inhibitors from spider venom (Table 2). Presented data let us distinguish three groups. Extremely active toxins with LD_50_ in the picomolar range, active toxins−LD_50_ in few nanomoles (this group includes ω-Tbo-IT1) and one moderately active toxin, which has LD_50_ more than 10 nmol/g. Cellular target and mode of action as seen from the table do not correlate with insect toxicity.

The toxin’s dosage in insects is in good agreement with measured activity on neuro-muscular junction in *C. vicina* (IC_50_ value of 40 ± 10 nM). Low IC_50_ value could have set a new toxin in line with candidate base bioinsecticides; however, its measured dose is inferior to one measured for “extremely active” toxins. Further point mutagenesis should remove this limitation because spatial structure data produce a good opportunity for modeling.

### Molecular target of action

Using different electrophysiology approaches, we demonstrated that ω-Tbo-IT1 acts as an inhibitor of insect Ca_v_ channels. Therefore, we compared ω-Tbo-IT1 with other calcium-channel inhibitors from spider venoms. More than 100 spider toxins acting on different calcium channels have already been described in scientific papers and deposited in the UniProt Database, (http://www.uniprot.org/). A few of them, however, were experimentally characterized in research papers as insect-selective calcium channel inhibitors. These are: ω-plectoxin-Pt1a (PLTX-II) from *Plectreurys tristis*^47^,^50^,^51^; ω-atracotoxin-Hv1a (ω-ACTX-Hv1a)^10^,^11^,^13^,^52^,^53^ and ω-atracotoxin-Hv2a (ω-ACTX-Hv2a) from *Hadronyche versuta*^54^; ω-atracotoxin-Ar1a (ω-ACTX-Ar1a) from *Athrax robustus*^10^; and huwentoxin-V (ω-TRTX-Hs2a)^55^,^56^.

Effect of ω-Tbo-IT1 demonstrates remarkable similarity with action of well-known toxin PLTX-II on *Drosophila* neuro-muscular junction^47^. Indeed, PLTX-II affected evoked, but not spontaneous, synaptic release. It was directly demonstrated that PLTX-II blocks Ca^2+^ currents in cultured embryonic *Drosophila* neurons^50^. More recent study suggests that PLTX-II selectively blocks the *Drosophila* Ca_v_2 channel (Dmca1A)^51^. Like ω-Tbo-IT1 in our experiments, PLTX-II has no effect on *Drosophila* muscle and it is inactive on synaptic transmission at frog neuromuscular junctions^50^. Summarizing foregoing effects affinity we assume that ω-Tbo-IT1 shares the same biological target as PLTX-II – insect Ca_v_2 channels.

In preparations of cockroach *P. americana* neurons we found that at least one type of Ca^2+^-currents in these neurons is strongly inhibited by ω-Tbo-IT1. It is known that four different kinds of Ca^2+^ currents were distinguished on the basis of their kinetics, voltage range of activation and pharmacological profile and can be evoked in dorsal unpaired median neurons isolated from abdominal ganglia of the American cockroach *P. americana*^57^. These currents are likely mediated by different types (or isoforms) of Ca_v_ channels (Ca_v_1, Ca_v_2 and Ca_v_3). Presumably the Ca_v_2 subtype of insect Ca^2+^ channels is sensitive to the toxin application.

The closest vertebrate analog of Ca_v_2 channels are N-type channels responsible for synaptic release of neurotransmitters, and so inhibitors of Ca_v_2 insect channel can potentially affect presynaptic N-type vertebrate channels. Therefore, action of ω-Tbo-IT1 was tested in several vertebrate synaptic preparations. Our electrophysiological experiments have demonstrated that the action was highly selective. This is not very surprising since sequence similarity between vertebrate and invertebrate channels is limited and the channels often differ by their sensitivity to pharmacological agents^58^. For instance, ω-ACTX-Hv1a strongly affects insect Ca_v_1 currents but has no effect on calcium currents in rat trigeminal neurons and rat Ca_v_1.2, Ca_v_2.1 and Ca_v_2.2 HVA channels^59^.

Certainly, we cannot exclude that ω-Tbo-IT1 can influence other channels. For instance, its action on sodium channels was not studied systematically.

### Structural comparison to ICK toxins

Among insect-selective calcium channel inhibitors from spider venoms, only for ω-atracotoxin-Hv1a and ω-atracotoxin-Hv2a spatial structures were identified^52^,^54^. In the most general terms, all three toxins resemble each other. They contain a globular hydrophobic core and a *С*-terminal tail that protrudes from it. ω-ACTX-Hv1a and ω-ACTX-Hv2a both contain three *N*-terminal residues extending beyond their central core that are very dynamic in solution^52^,^54^. In contrast, ω-Tbo-IT1 starts straight from the hydrophobic core. We think that short flexible *N*-terminal regions in ω-ACTX-Hv1a and ω-ACTX-Hv2a hardly influence activity of toxins.

The *C*-terminal region of ω-ACTX-Hv1a is a finger-like β-hairpin that is formed by residues 22–37^52^. It doesn’t share primary structure homology to the same region of ω-Tbo-IT1 but is identical to it by backbone fold (Fig. 7C). Two anti-parallel β-strands in the hairpin are stabilized not only by hydrogen bonds but also one disulfide bond, in the case of ω-Tbo-IT1.

Despite a good alignment of the structures of ω-Tbo-IT1 and ω-ACTX-Hv1a, the major conformation of ω-Tbo-IT1 has an inverse direction of the polypeptide chain in the tip of the major β-hairpin loop due to different local structure of the bulge regions of the β-strands. Nevertheless, the ω-Tbo-IT1 minor conformation’s twisted major β-hairpin loop and β-hairpin loop of ω-ACTX-Hv1a are comparable. The β-hairpin in ω-Tbo-IT1 core has an unusual link between two strands, which is not a short β-turn (as usual in ICK-toxins), but γ-turn instead. Notably, their tips are alternatively charged, ω-Tbo-IT1 has two positive residues Arg32 and Lys33, whereas in contrast ω-ACTX-Hv1a has negative residues Glu26 and Glu28.

For ω-ACTX-Hv1a it was shown experimentally that β-hairpin is indispensable for insecticidal activity. Thus, a β-hairpinless mutant of this toxin is devoid of insecticidal activity^60^, and two residues Asn27 and Arg35 together with Pro10 are essential for activity^59^. For ω-ACTX-Hv2a it was experimentally revealed by truncation that *C*-terminal β-hairpin is also indispensable for an activity like the one in ω-ACTX-Hv1a^54^. By analogy we assume that the following residues in ω-Tbo-IT1 may be involved in binding: Asn10, Arg32 and/or Lys33.

As it was mentioned above, the small β-hairpin loop with 3_10_-helical turn is absent in ω-ACTX-Hv1a since its Cys II-Cys III interval is shorter by 6 residues. Our hypothesis is that the short β-hairpin helps major *C*-terminal β-hairpin during the process of interaction of ω-Tbo-IT1 with the channel. This speculation might be fortified by some NMR signal doubling and broadening (Supplementary Figs S1 and S2) we observed (e.g. for Thr15 and Thr29 from short β-hairpin and major β-hairpin, correspondingly). Therefore, we observed minor cross peak signals, which may imply simultaneous movements of two hairpins (these movements are shown by arrows in Fig. 7C). Moreover, we hypothesize here that twist- and scissors-like motions might be coordinated, because we observed formation of additional hydrogen bonds, namely those between residues Asn36-Tyr13 and Asn36-Thr15 correspondingly. Such formation would be impossible if motions under consideration could have been non-simultaneous. Postulated β-hairpin loop movements in ω-Tbo-IT1 may serve for searching the proper binding site in the target calcium channel.

## Conclusion

In summary, work discussed describes structural and electrophysiological characterization of novel inhibitor of insect calcium channels (presumably, insect Ca_v_2 type). This inhibitor is a toxin, by primary structure related to some sodium channel inhibitors, but its target is neuronal insect voltage-gated Ca^2+^ channels. We suspect that the interaction of ω-Tbo-IT1 with channels is mediated by twist and scissors-like motions of two β-hairpin loops. Loop movement results in distance variation between the ‘hot spots’ of the toxin-receptor binding and facilitate interaction. Such ‘dynamic epitope’ may be regarded as an evolutionary step forward from the static epitope.

## Additional Information

**How to cite this article**: Mikov, A. N. *et al.* ω-Tbo-IT1 - New Inhibitor of Insect Calcium Channels Isolated from Spider Venom. *Sci. Rep.* **5**, 17232; doi: 10.1038/srep17232 (2015).

## Supplementary Material

Supplementary Information

## Figures and Tables

**Table 1:** Structural statistics for the ensemble of 20 best NMR structures of ω-Tbo-IT1.

NMR structure (PDB code)	code in progress
Number of NMR distance & dihedral restraints	Statistic
Total unambiguous upper NOE restraints	422
intra-residue (|i-j| = 0)	249
inter-residue	173
sequential (|i-j| = 1)	103
medium-range (1 < |i-j| < 4)	21
long-range (|i-j| > 4)	49
Total lower restraints (missing NOE)	12816
Hydrogen bond restraints, upper/lower (bonds)	44/44 (22)
SS-bond restraints, upper/lower (bonds)	12/12 (4)
Total torsion angle restraints, intervals (angles)	178 (64)
backbone φ, intervals (angles)	89 (32)
side chain χ^1^, intervals (angles)	89 (32)
Structure calculation statistics	
CYANA target function (Å^2^)	1.03 ± 0.17
Restraint violations	
upper distance (>0.2 Å, >0.3 Å)	2 ± 1, 0 ± 1
lower distance limits(>0.2 Å)	0 ± 1
dihedral (>5°)	0
Average pairwise RMSD (Å)	
backbone atoms	0.53 ± 0.14
all heavy atoms	1.49 ± 0.19
Ramachandran analysis£	
% residues in most favored regions	66.6
% residues in additional allowed regions	33.1
% residues in generously allowed regions	0.3†
% residues in disallowed regions	0.0†

^£^Ramachandran statistics was determined using CYANA^33^.

^†^Residues from unfolded and flexible regions.

**Table 2:** Insecticidal relevance of ω-Tbo-IT1.

	Toxin	Effect	Spider species	Insect tested	LD_50_, nmol/g	Link
#1	μ-Aga IV	μ	*Agelenopsis aperta*	*Musca domestica*	0.03	61
#2	Tx4(6-1)	δ	*Phoneutria nigriventer*	*Musca domestica*	0.04	62
#3	ω-ACTX-Hv1a	ω	*Hadronyche versuta*	*Musca domestica*	0.08	42
#4	ω-ACTX-Ar1a	ω	*Atrax robustus*	*Acheta domesticus*	0.14	10
#5	ω-ACTX-Hv2a	ω	*Hadronyche versuta*	*Acheta domesticus*	0.16	54
#6	μ-Aga II*	μ	*Agelenopsis aperta*	*Musca domestica*	1.38	61
#7	δ-PaluIT1*	δ	*Pireneitega luctuosa*	*Spodoptera litura*	2.35	43
#8	HWTX-V	ω	*Haplopelma schmidti*	*Locusta migratoria*	3.89	55
#9	ω-Tbo-IT1	ω	*Tibellus oblongus*	*Musca domestica*	4.40	this paper
#10	δ-PaluIT4*	δ	*Pireneitega luctuosa*	*Spodoptera litura*	11.03	43

Most powerful insect-selective ICK toxins from spider venom and three toxins with a best similarity by pBLAST are compared. Type of molecular target for toxins written in the effect column where: (μ) indicates a shift of voltage-dependent activation of sodium channel; (δ) effect on sodium channel inactivation; (ω) interaction with calcium channel. * the best homology to ω-Tbo-IT1 by pBLAST.
