# Supplementary material for: ω-Tbo-IT1–New Inhibitor of Insect Calcium Channels Isolated from Spider Venom
Source: Sci Rep. 2015 Nov 27;5:17232. doi: 10.1038/srep17232 (PMC4661699; doi:10.1038/srep17232)
Supplement: Supplementary Information [file srep17232-s1.pdf]

Supplementary information

ω-Tbo-IT1 – New Inhibitor of Insect Calcium Channels Isolated from Spider Venom

Alexander N. Mikov[1], Irina M. Fedorova[2], Natalia N. Potapieva[2], Ekaterina E. Maleeva[1], Yaroslav A. Andreev[1], Eduard V. Bocharov[3, 4], Timur N. Bozin[4], Dmitry A. Altukhov[4], Alexey V. Lipkin[4], Sergey A. Kozlov[1], Denis B. Tikhonov[2], Eugene V. Grishin[1]

[1] Department of Molecular Neurobiology, Shemyakin–Ovchinnikov Institute of Bioorganic Chemistry RAS, Russia

[2] Laboratory of Biophysics of Synaptic Processes, I.M. Sechenov Institute of Evolutionary Physiology and Biochemistry RAS, Russia

[3] Department of Structural Biology, Shemyakin–Ovchinnikov Institute of Bioorganic Chemistry RAS, Russia

[4] NBIC Centre, NRC "Kurchatov Institute", Russia

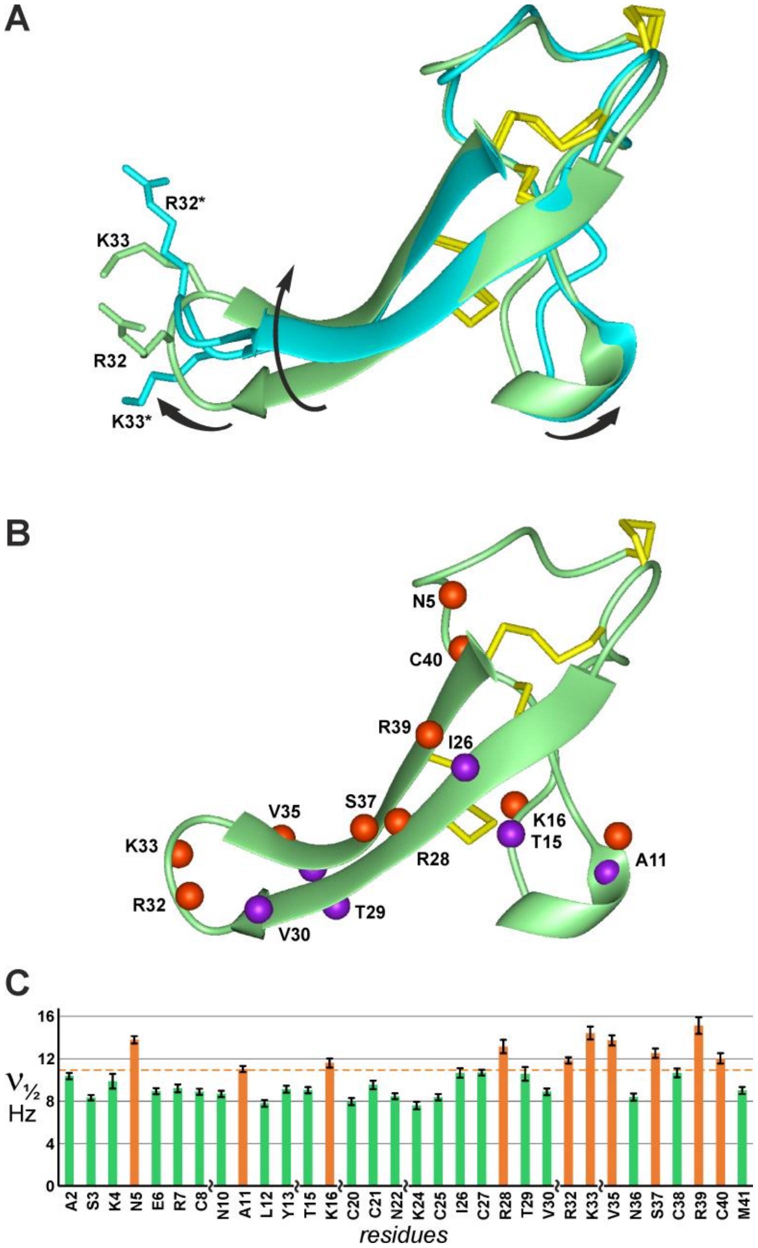

Supplementary Fig. S1. Relaxation NMR data and conformational exchange in ω-Tbo-IT1. (A) Spatial structure alignment of the major (in *green*) and minor (in *cyan*) conformations of ω-Tbo-IT1. The arrows indicate the postulated twist and scissors-like mutual motions of the β-hairpin loops of ω-Tbo-IT1. The side-chains of the residues alternatively charged the tips of ω-Tbo-IT1 (positive R32/R32* and K33/K33* for major/minor conformations) are shown. (B) Ribbon representation of major conformation of ω-Tbo-IT1. Residues of ω-Tbo-IT1 exhibiting pronounced broadening of backbone amide ^{1}H resonances (having enhanced R_{ex}, see panel C), which indicate slow conformational exchange in micro-millisecond time scale, are shown by *orange* balls (H^{N} atoms). Residues of ω-Tbo-IT1exhibiting disproportional doubling of methyl cross-peak in ^{1}H-^{13}C HSQC spectrum (see Supplementary Fig. S2) are shown by *purple* balls (Cα atoms). (C) Linewidths $\nu_{1/2}$ (width at half maximum of the peak) of the ^{1}H resonances of ω-Tbo-IT1 amide groups. The linewidths were derived from ^{1}H projections of the 2D amide cross-peaks (excluding Gly and Pro residues) in the NOESY spectrum of ω-Tbo-IT1 *via* fitting by Lorentzian lines with Mathematica software (Wolfram Research, U.S.A.) taking into account the $^{3}J_{HNH\alpha}$ coupling constants. The linewidths $\nu_{1/2}$ are proportional to the observed apparent transverse relaxation rates $R_2^{Obs} = R_2^{0} + R_{ex}$, where R_2^{0} is the intrinsic relaxation rate and R_{ex} reflects additional line broadening due to intermediate conformational exchange in micro-millisecond time scale (Cavanagh et al, *Protein NMR Spectroscopy*. *Protein NMR Spectroscopy*, Elsevier, 2007). The amide ^{1}H resonances having enhanced $\nu_{1/2}$ more than 11 Hz (that is definitely above than average value equal to 10.4 Hz) are highlighted in *orange*. The uncertainties are shown by bars.

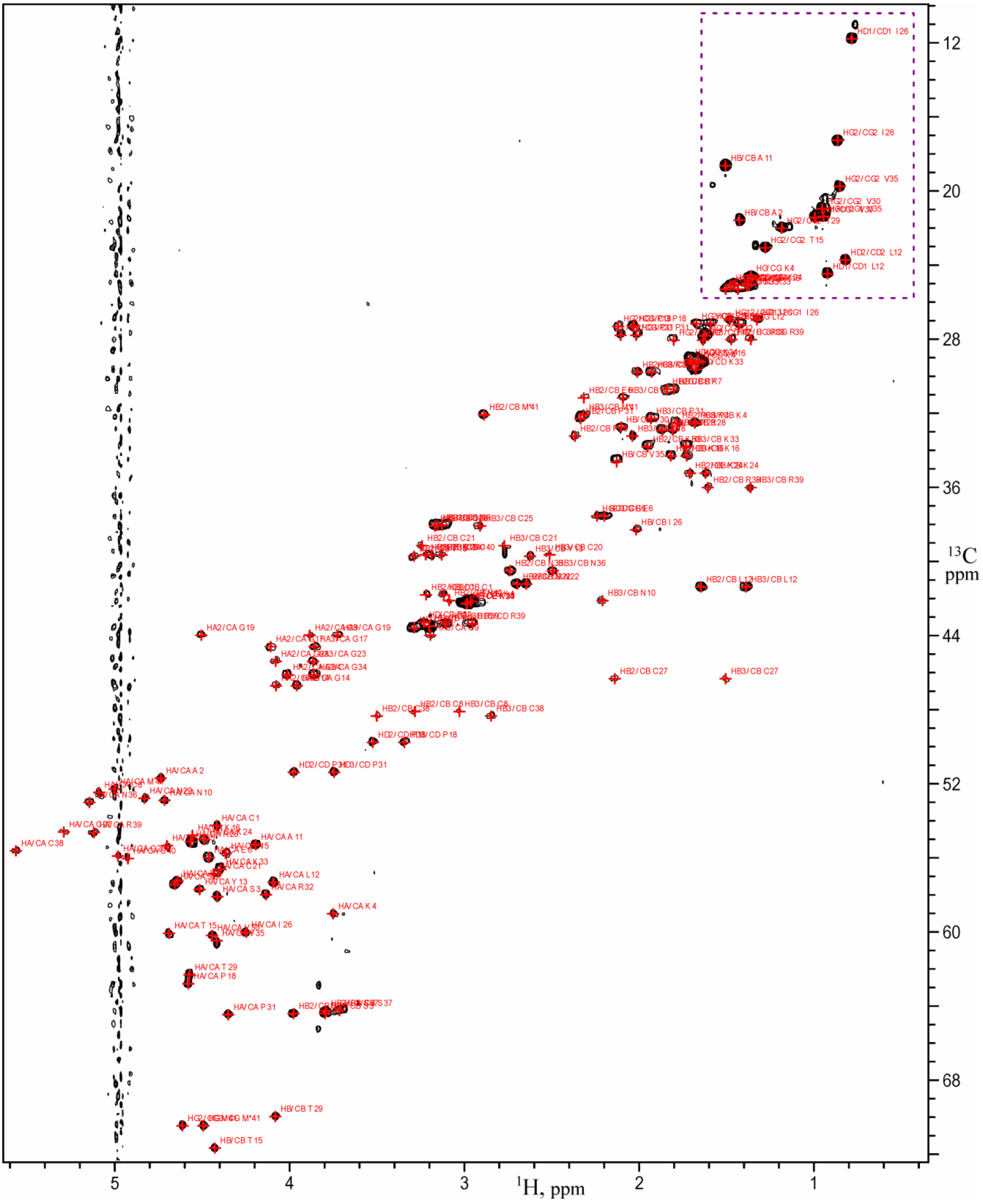

Supplementary Fig. S2. Two-dimensional heteronuclear ^{1}H-^{13}C HSQC (at natural abundance) NMR spectra acquired at 12°C for 1.5 mM recombinant ω-Tbo-IT1 toxin solubilized in H_2O with 20 mM phosphate buffer, pH 5.8. Cross-peaks assignments of CH, CH_2 and CH_3 groups of major conformation of ω-Tbo-IT1 are indicated (adapted from CARA program). The methyl cross-peak region, where broadening and disproportional peak doubling are well observed, is highlighted by dashed *purple* line.

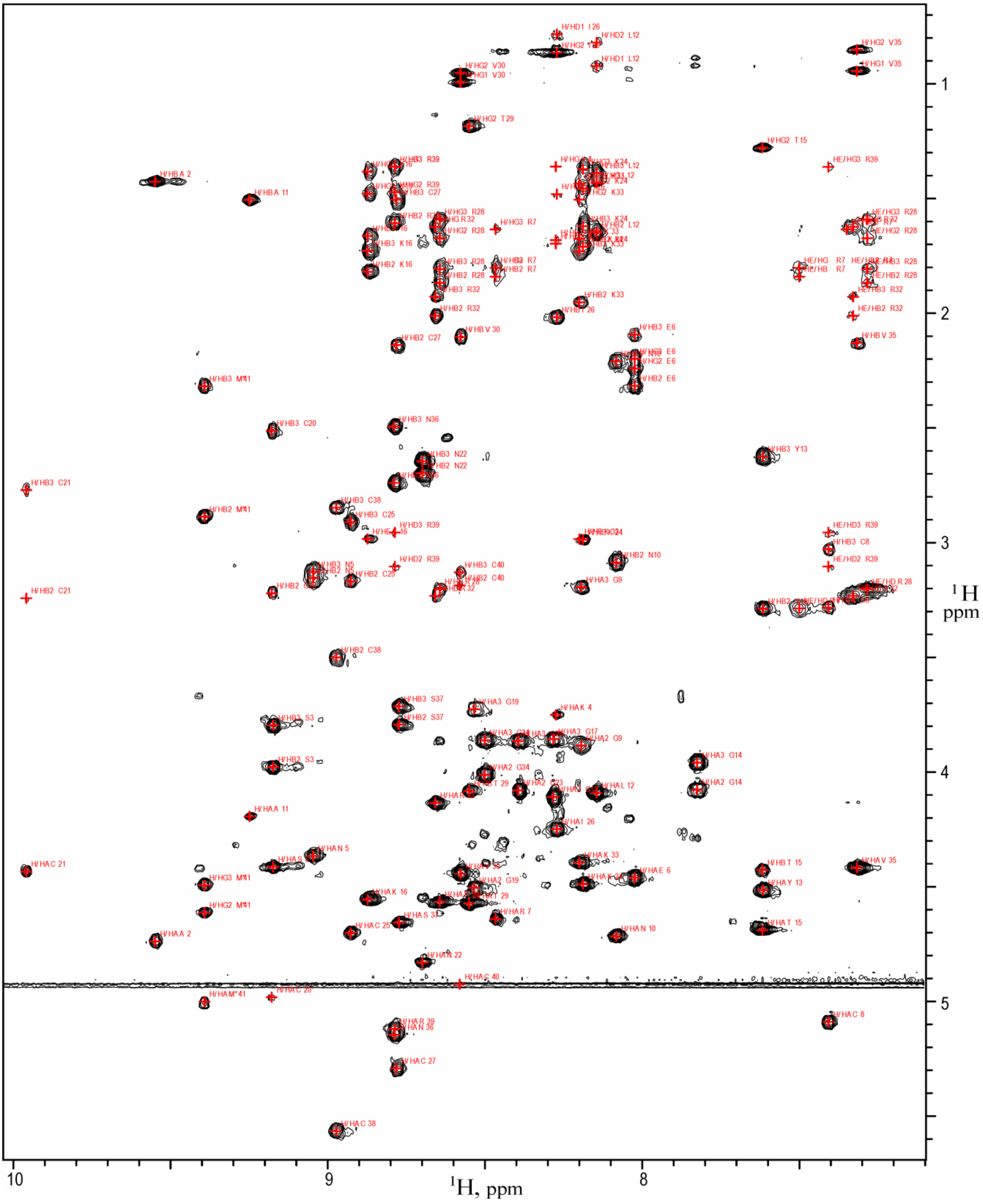

Supplementary Fig. S3. Fragment of the ^{1}H-^{1}H TOCSY NMR spectrum (see Supplementary Fig. S4*A*) with the amide cross-peaks assignments of major conformation of ω-Tbo-IT1 (adapted from CARA program).

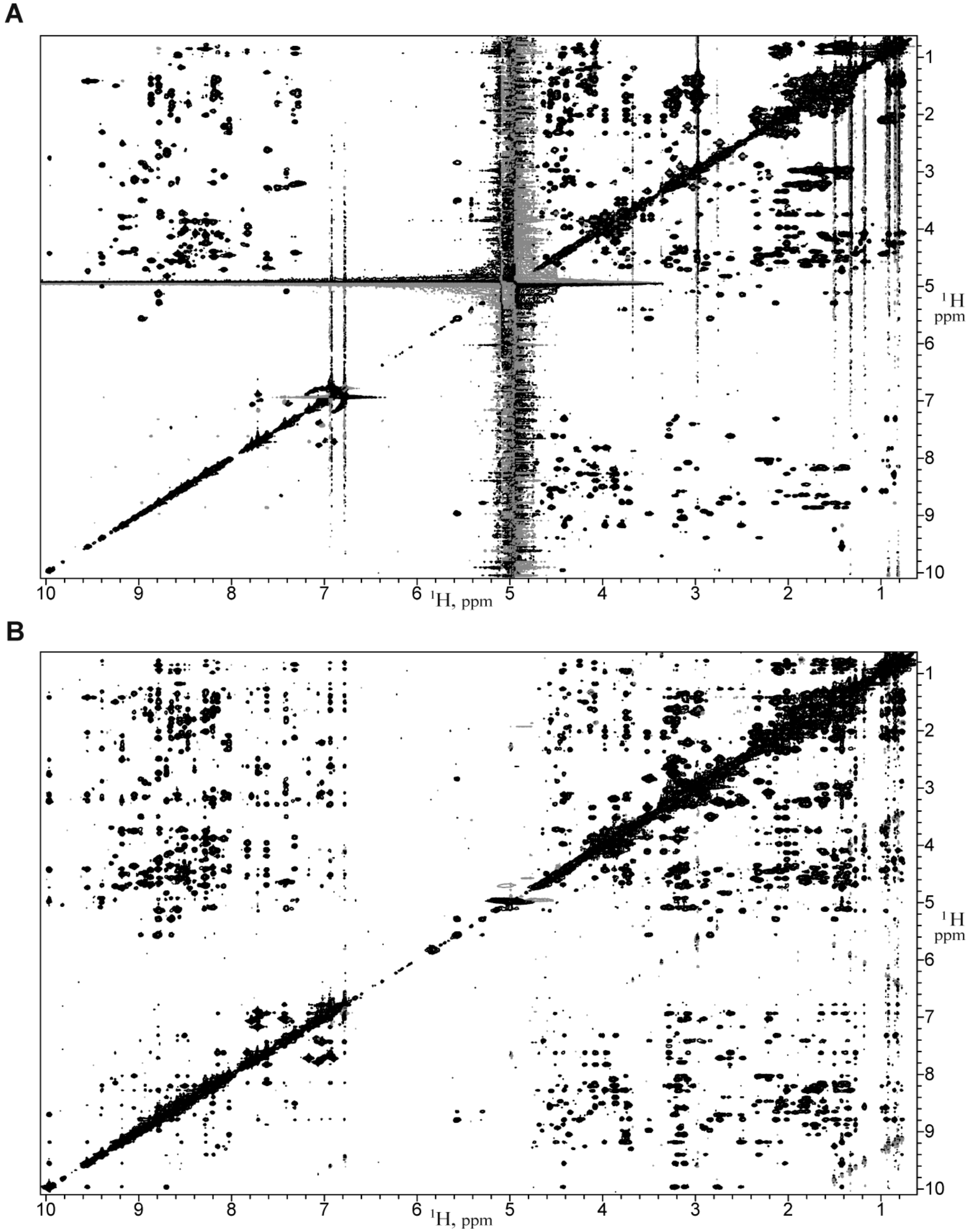

Supplementary Fig. S4. Two-dimensional (A) ^{1}H-^{1}H TOCSY (80-ms mixing time) and (B) NOESY (150-ms mixing time) NMR spectra acquired at 12°C for 1.5 mM recombinant ω-Tbo-IT1 toxin solubilized in H_2O with 20 mM phosphate buffer, pH 5.8.

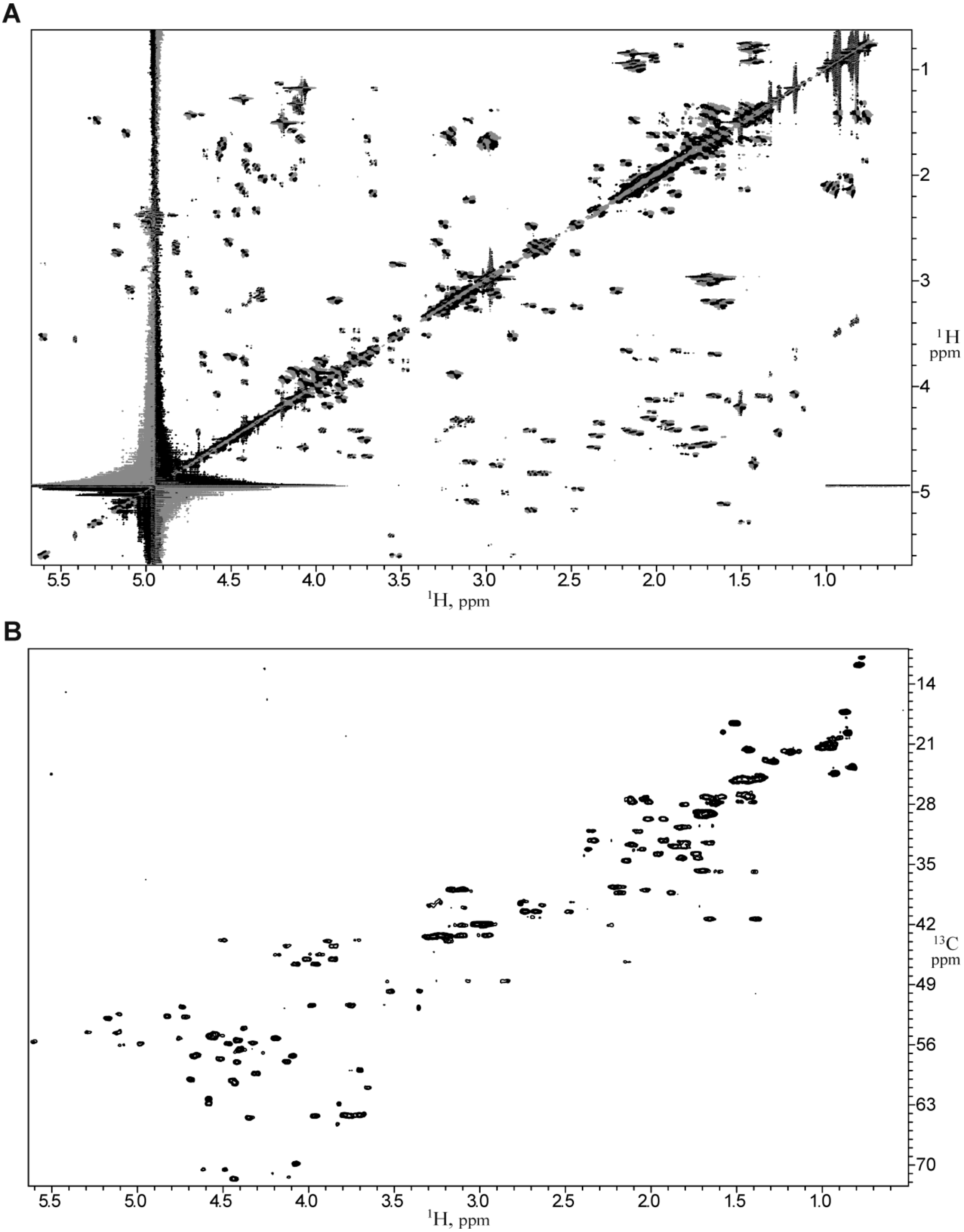

Supplementary Fig. S5. Two-dimensional (A) ^{1}H-^{1}H DQF-COSY (Z-gradient version) and (B) heteronuclear ^{1}H-^{13}C HSQC (at natural abundance) NMR spectra acquired at 12°C for 1.5 mM recombinant ω-Tbo-IT1 toxin solubilized in D_2O with 20 mM phosphate buffer, pH 5.8.

Supplementary information - PSVS

ω-Tbo-IT1 – New Inhibitor of Insect Calcium Channels Isolated from Spider Venom

Alexander N. Mikov[1], Irina M. Fedorova[2], Natalia N. Potapieva[2], Ekaterina E. Maleeva[1], Yaroslav A. Andreev[1], Eduard V. Bocharov[3, 4], Timur N. Bozin[4], Dmitry A. Altukhov[4], Alexey V. Lipkin[4], Sergey A. Kozlov[1], Denis B. Tikhonov[2], Eugene V. Grishin[1]

[1]Department of Molecular Neurobiology, Shemyakin–Ovchinnikov Institute of Bioorganic Chemistry RAS, Russia

[2]Laboratory of Biophysics of Synaptic Processes, I.M. Sechenov Institute of Evolutionary Physiology and Biochemistry RAS, Russia

[3]Department of Structural Biology, Shemyakin–Ovchinnikov Institute of Bioorganic Chemistry RAS, Russia

[4]NBIC Centre, NRC "Kurchatov Institute", Russia

Protein Structure Validation Suite (PSVS)

PSVS report for TboIT1

Table of Contents

PSVS report for TboIT1 ..... **1**

Software Environment **2**
- Software for structure quality evaluation: 2
- MolProbity programs: 2
- Other Software: 2

Structure Quality Analysis for NAME **3**

Summary of structure quality factors **9**

Detailed results of TboIT1 by PSVS **10**
- Output from PDBStat 10
  - S(phi)|S(psi) V/S Residue number 10
  - Table of Backbone and Heavy Atom RMSD 11
- Output from PROCHECK 13
  - Ramachandran Plot for all models 13
  - Residue Properties for all models 15
  - Model Secondary Structures from Procheck 15
  - Ramachandran Plots for each residue 18
  - Ramachandran analysis for each residue from Molprobity 20
  - Chi1-Chi2 Plots for each residue 20
  - Procheck G-factors for phi-psi for each residue 22
  - Procheck G-factors for all dihedral angles for each residue 23
- Output from Verify3D 24
  - Verify3D Score over a window of $winsize_s residues 24
- Output from ProsaII 24
  - ProsaII Score over a window of $winsize_s residues 24
- Output from MolProbity 25
  - VdW violations from MAGE 25
- Output from PDB validation software 31
  - Summary from PDB validation 31

PSVS report for TboIT1

Protein Structure Validation Suite (PSVS)

Software Environment

Software for structure quality evaluation:

```
DSSP            DsspCMBI-April-2000
pdbstat         PdbStat-5.9 Version
AutoAssign      Version 2.4.0 (uses only AVS scripts)
RPF analysis    ASDP-1.0
PDB validation  Version 8.061
Verify3D        Version 1.0 corrected by Aneerban
ProsaII         Prosa2003
PROCHECK        Version 3.5.4
MolMol          Version 2K.2
```

MolProbity programs:

```
        cluster           1999
        clashlistcluster  1999 (corrected by Aneerban)
        mage              Version 6.35.040409
        prekin            Version 6.35.040406
        reduce            Version 2.14
        probe             Version 2.6
```

Other Software:

```
PERL           Version 5.8.0
convert        ImageMagick 5.5.6
ps2pdf         Ghostscript 7.05
htmldoc        v1.9
gnuplot        Version 3.7 patchlevel 3
jpegtopnm      year 2000
pnmcrop        year 2000
pnmtojpeg      year 2000
```

Structure Quality Analysis for NAME

Analyses performed for all residues.
Procheck analysis,RMSD calculation and structure superimposition are based on: all residues

NESG ID:	NAME
PDB ID:	
Deposition date:	
Common Name:	
Class:	
Length (a.a.):	41
Organism:	
SwissProt / TrEMBL ID:	
# models:	20
Oligomerization:	monomer
Molecular weight:	4340

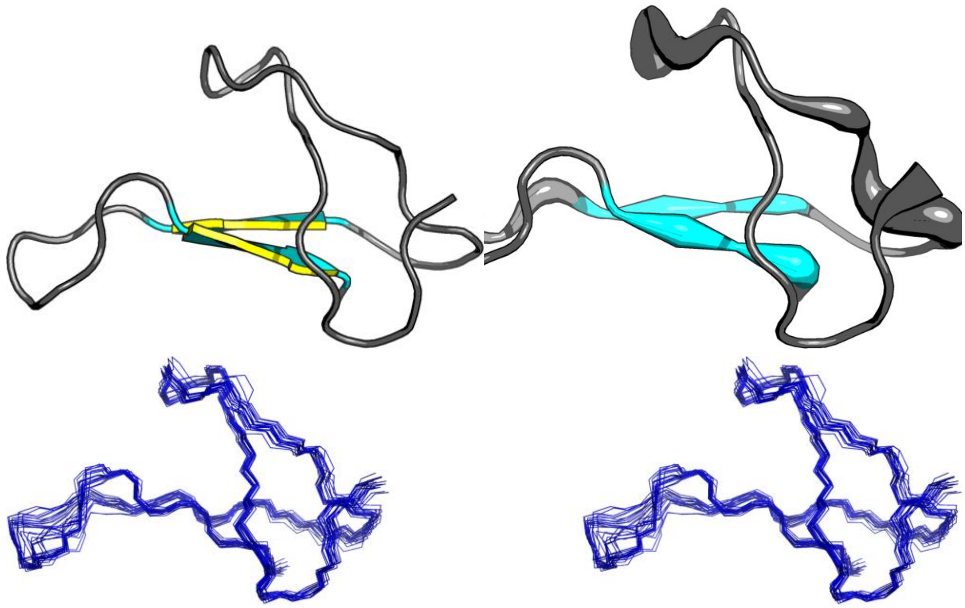

Secondary Structure Elements:
alpha helices:
beta strands: 25A-30A, 34A-40A

FIDs deposited in the BMRB? no

RMSD	*All residues*	*Ordered residues*[2]	*Selected residues*[3]
All backbone atoms	0.6 Å	0.6 Å	0.6 Å
All heavy atoms	1.3 Å	1.3 Å	1.3 Å

Ramachandran Plot Summary for selected residues[3] from Procheck

Most favoured regions	*Additionally allowed regions*	*Generously allowed regions*	*Disallowed regions*
67.3%	31.9%	0.8%	0.0%

Ramachandran Plot Summary for selected residues[3] from Richardson Lab's Molprobity

Most favoured regions	*Allowed regions*	*Disallowed regions*	View plot	View model summary
87.3%	11.8%	0.9%		

Global quality scores

Program	*Verify3D*	*ProsaII (-ve)*	*Procheck (phi-psi)*[3]	*Procheck (all)*[3]	*MolProbity Clashscore*
-Raw score	0.33	0.33	-0.92	-0.81	9.66
Z-score[1]	-2.09	-1.32	-3.30	-4.79	-0.13

Close Contacts and Deviations from Ideal Geometry (from PDB validation software)

Number of close contacts (within 1.6 &Aring for H atoms, 2.2 &Aring for heavy atoms): 0

RMS deviation for bond angles:	0.2 °
RMS deviation for bond lengths:	0.001 Å

[1] With respect to mean and standard deviation for a set of 252 X-ray structures < 500 residues, of resolution <= 1.80 Å, R-factor <= 0.25 and R-free <= 0.28; a positive value indicates a 'better' score
[2]Order residues: HASH(0xd7f2a0)
[3]Selected residues: all

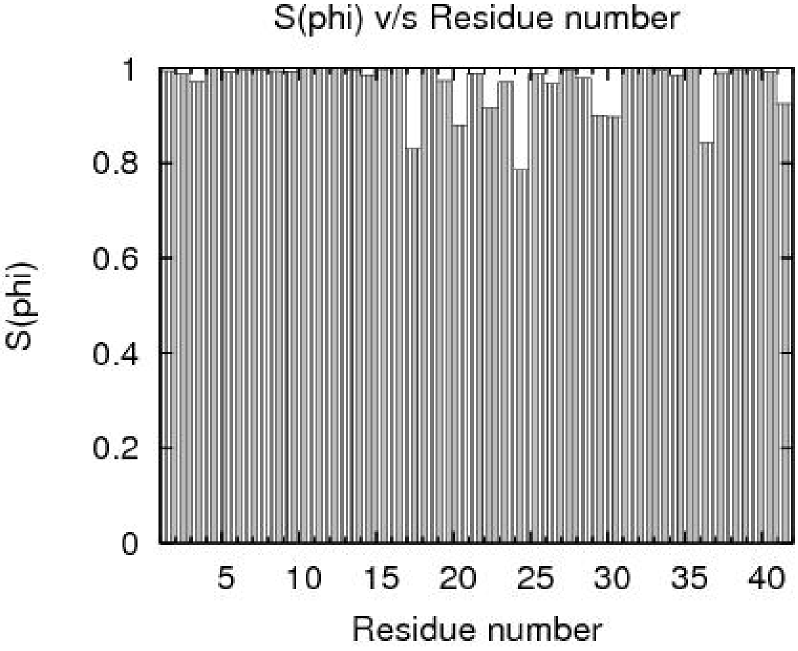

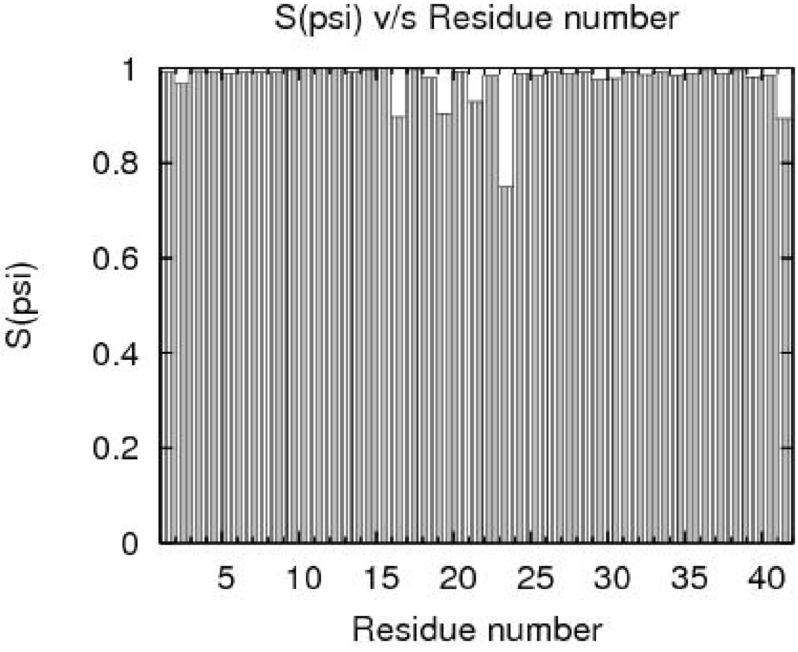

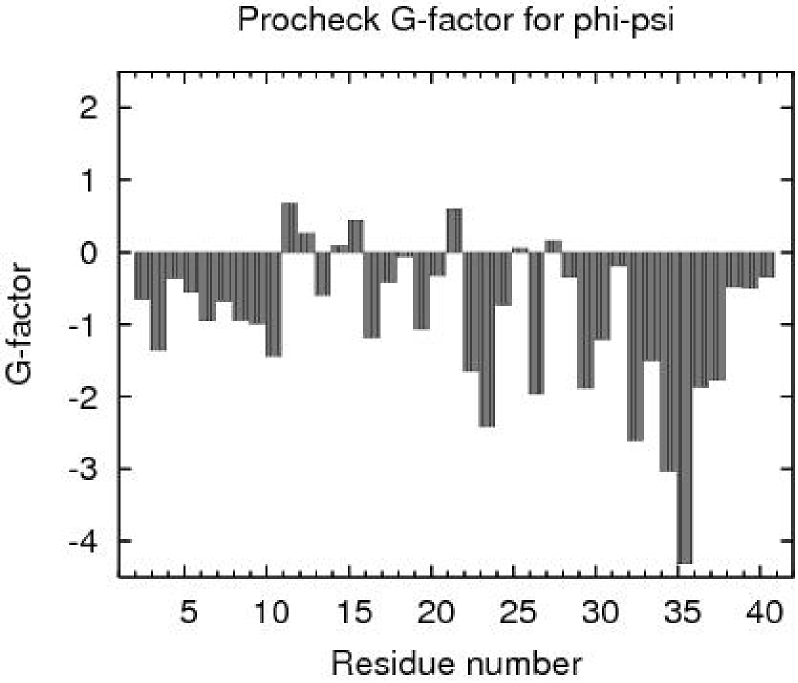
Procheck G-factor for phi-psi
G-factor
2
1
0
-1
-2
-3
-4
5
10
15
20
25
30
35
40
Residue number

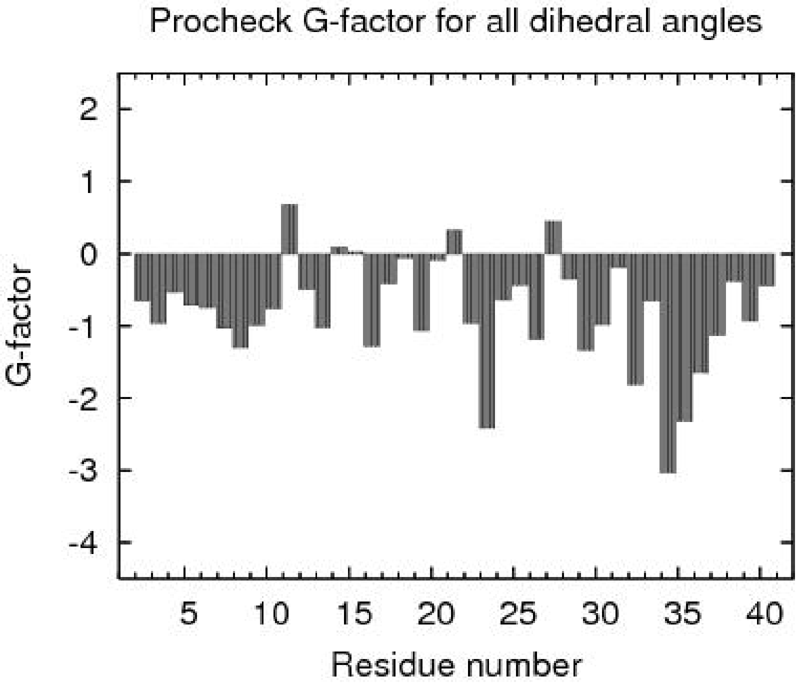
Procheck G-factor for all dihedral angles
G-factor
2
1
0
-1
-2
-3
-4
5
10
15
20
25
30
35
40
Residue number

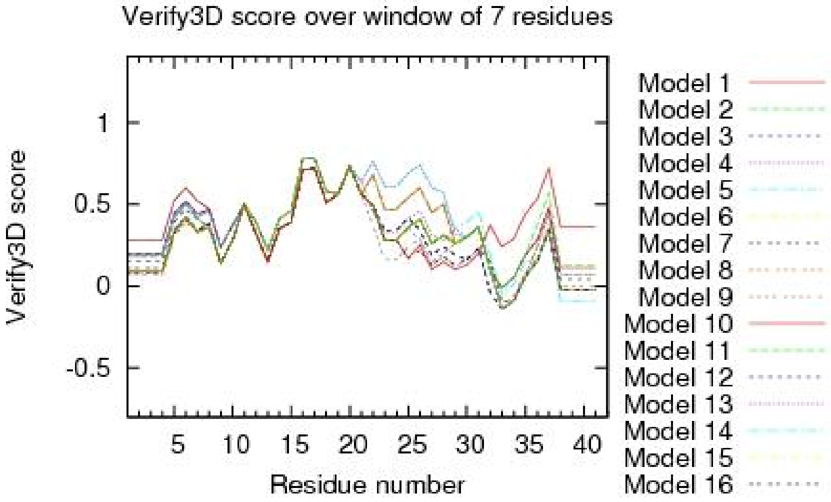
Verify3D score over window of 7 residues
Verify3D score
1
0.5
0
-0.5
5 10 15 20 25 30 35 40
Residue number
Model 1
Model 2
Model 3
Model 4
Model 5
Model 6
Model 7
Model 8
Model 9
Model 10
Model 11
Model 12
Model 13
Model 14
Model 15
Model 16

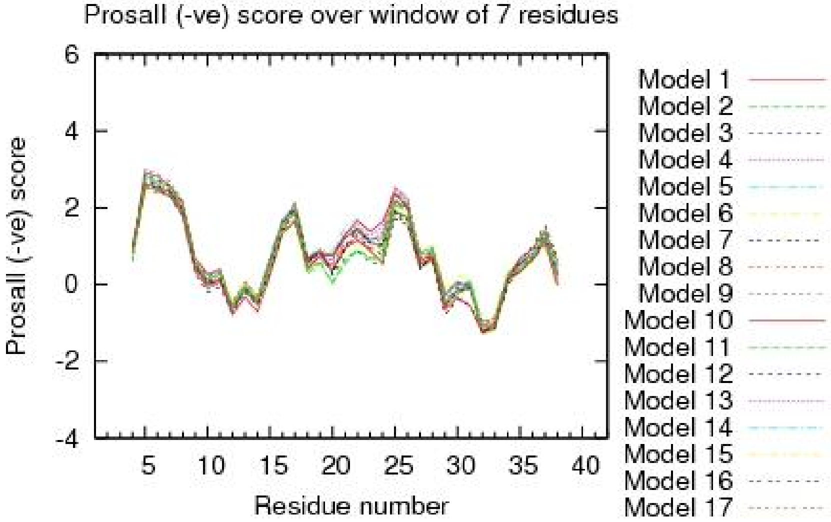
Prosall (-ve) score over window of 7 residues
Prosall (-ve) score
6
4
2
0
-2
-4
5 10 15 20 25 30 35 40
Residue number
Model 1
Model 2
Model 3
Model 4
Model 5
Model 6
Model 7
Model 8
Model 9
Model 10
Model 11
Model 12
Model 13
Model 14
Model 15
Model 16
Model 17

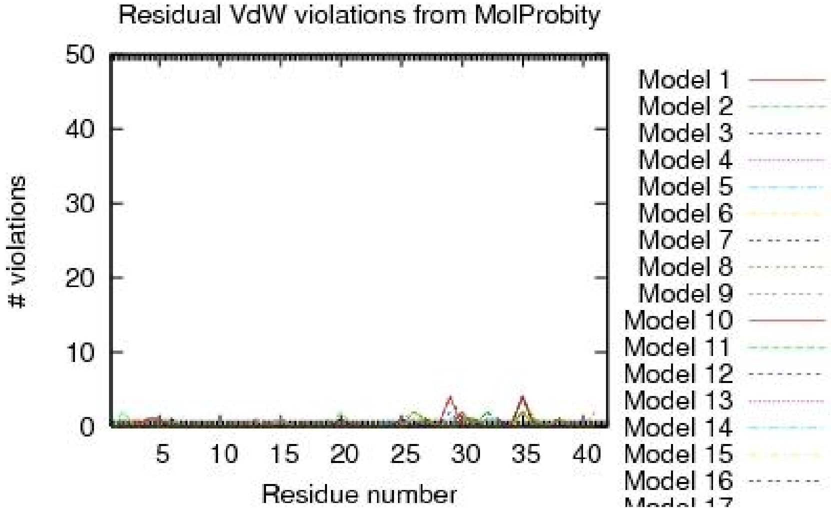
Residual VdW violations from MolProbity
violations
50
40
30
20
10
0
5 10 15 20 25 30 35 40
Residue number
Model 1
Model 2
Model 3
Model 4
Model 5
Model 6
Model 7
Model 8
Model 9
Model 10
Model 11
Model 12
Model 13
Model 14
Model 15
Model 16

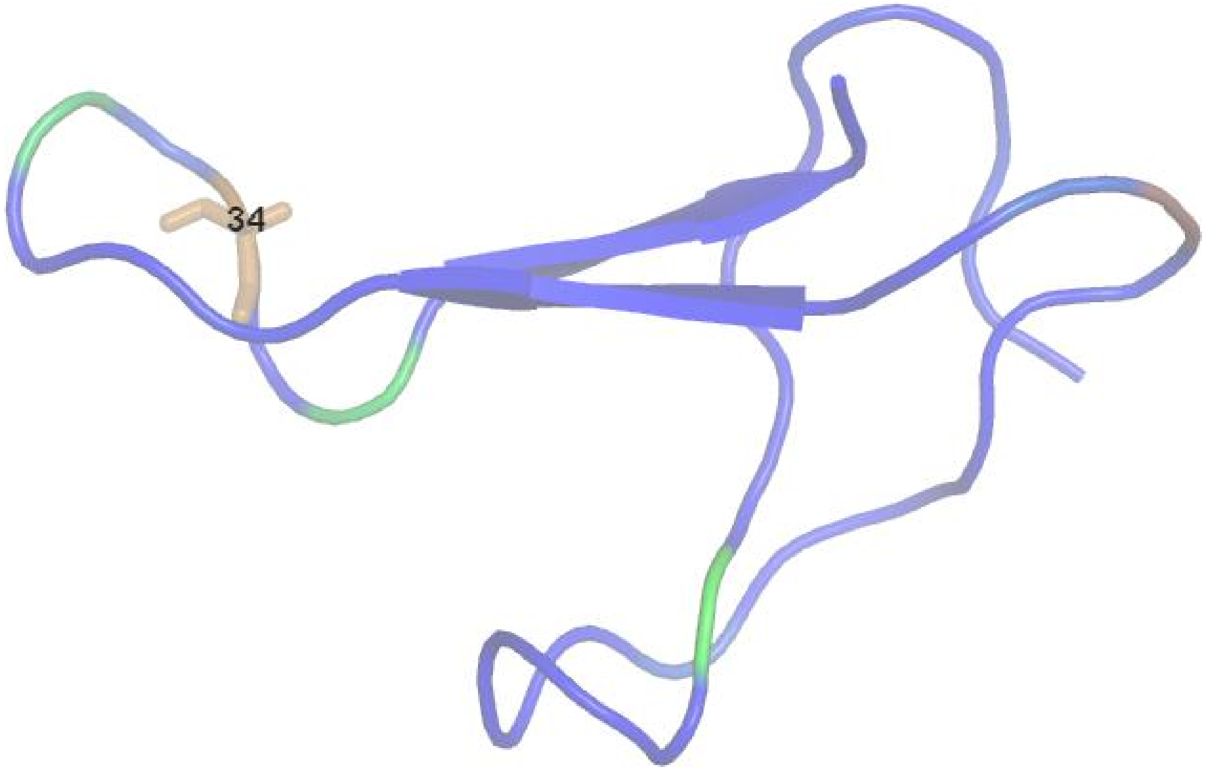

Residue Plot of Ramachandran anlysis(based on data from Richardson Lab's Molprobity)

References:

1. Luthy R, Bowie J U and Eisenberg D, "Assessment of protein models with three-dimensional profiles", Nature 356 (1992): 83-85
2. Bowie J U, Luthy R and Eisenberg D, "A Method to Identify Protein Sequences that Fold into a Known Three-Dimensional Structure", Science 253 (1991): 164-169
3. Sippl M J, "Recognition of Errors in Three-Dimensional Structures of Proteins", Proteins 17 (1993): 355-362
4. Sippl M J, "Calculation of Conformation Ensembles from Potentials of Mean Force", J Mol Biol 213 (1990): 859-883
5. Laskowski R Ai et al, "AQUA and PROCHECK_NMR: Programs for checking the quality of proteins structures solved by NMR", J Biomolec NMR 8 (1996): 477-486
6. Laskowski R A et al "PROCHECK: a program to check the stereochemical quality of protein structures" J Appl Cryst, 26 (1993): 283-291
7. Word J M et al, "Exploring steric constrains on protein mutations using MAGE / PROBE", Prot Sci 9 (2000): 2251-2259
8. Word J M et al, "Asparagine and Glutamine: Using Hydrogen Atom Contacts in the Choice of Side-chain Amide Orientation", J Mol Biol 285 (1999): 1735-1747
9. Word J M et al, "Visualizing and Quantifying Molecular Goodness-of-Fit: Small-probe Contact Dots with Explicit Hydrogens", J Mol Biol 285 (1999): 1711-1733
10. Tejero R and Montelione G T, "PDBStat", unpublished
11. Luthy R, McLachlan A D and Eisenberg D, "Secondary Structure-Based Profiles: Use of Structure-Conserving Scoring Tables in Searching Protein Sequence Databases for Structural Similarities", Proteins 10 (1991): 229-239
12. Richardson D C, Richardson J S, "The kinemage: a tool for scientific communication", Prot Sci 1(1) (1992): 3-9
13. Koradi, R, et al, "MOLMOL: a program for display and analysis of macromolecular structures ", J Mol Graphics 14 (1996): 51-55.

14. Güntert, P, Mumenthaler, C & Wüthrich, K "Torsion angle dynamics for NMR structure calculation with the new program DYANA", J. Mol. Biol 273 (1997): 283-298
15. Lovell S C et al, "Structure validation by Calpha geometry: phi,psi and Cbeta deviation" Proteins (2003) 50: 437-450
16. Kabsch W, Sander C, "Dictionary of protein secondary structure: pattern recognition of hydrogen-bonded and geometrical features", Biopolymers (1983) 22: 2577-2637
17. Bagaria, A., Jaravine, V., Huang, Y.J., Montelione, G.T., and Guntert, P. "Protein structure validation by generalized linear model root-mean-square deviation prediction". Protein Sci 21(2012), 229-238.

Summary of structure quality factors

Analyses performed for all residues.

Total structures computed	currently unknown		
Number of structures used	20		
RMSD Values			
	all	ordered[e]	Selected[f]
All backbone atoms	0.6 Å	0.6 Å	0.6 Å
All heavy atoms	1.3 Å	1.3 Å	1.3 Å
Structure Quality Factors - overall statistics			
	Mean score	SD	Z-score [g]
Procheck G-factor [e] (phi / psi only)	-0.92	N/A	-3.30
Procheck G-factor [e] (all dihedral angles)	-0.81	N/A	-4.79
Verify3D	0.33	0.0301	-2.09
ProsaII (-ve)	0.33	0.0357	-1.32
MolProbity clashscore	9.66	2.7335	-0.13
Ramachandran Plot Summary from Procheck [f]			
Most favoured regions	67.3%		
Additionally allowed regions	31.9%		
Generously allowed regions	0.8%		
Disallowed regions	0.0%		
Ramachandran Plot Statistics from Richardson's lab			
Most favoured regions	87.3%		
Allowed regions	11.8%		
Disallowed regions	0.9%		

[e] Residues with sum of phi and psi order parameters > 1.8

Ordered residue ranges:
[f] Residues selected based on: all residues

Selected residue ranges: all

[g] With respect to mean and standard deviation for for a set of 252 X-ray structures < 500 residues, of resolution <= 1.80 Å, R-factor <= 0.25 and R-free <= 0.28; a positive value indicates a 'better' score

Generated using PSVS 1.5

Detailed results of TboIT1 by PSVS

Output from PDBStat

S(phi)|S(psi) V/S Residue number

Text output from PDBStat of phi psi order

#	CHAIN										.GT.	SUM.GT.
#	RES	ID	DIH	S(phi)	S(psi)	S(chi1)	S(chi2)	S(chi3)	S(chi4)	S(chi5)	0.90	1.6
	CYS	A	1		0.994	0.992						
	ALA	A	2	0.988	0.968						2	2
	SER	A	3	0.972	0.994	0.261					3	3
	LYS	A	4	0.999	0.992	0.360	0.788	0.689	0.427		4	4
	ASN	A	5	0.992	0.989	0.620	0.754				5	5
	GLU	A	6	0.996	0.992	0.784	0.539	0.141			6	6
	ARG	A	7	0.997	0.992	0.380	0.756	0.454	0.370	1.000	7	7
	CYS	A	8	0.994	0.991	0.981					8	8
	GLY	A	9	0.993	0.996						9	9
	ASN	A	10	1.000	0.999	1.000	0.990				10	10
	ALA	A	11	1.000	0.999						11	11
	LEU	A	12	1.000	1.000	0.618	0.743				12	12
	TYR	A	13	0.997	0.992	0.999	0.045				13	13
	GLY	A	14	0.985	0.997						14	14
	THR	A	15	0.998	1.000	0.969					15	15
	LYS	A	16	0.999	0.898	0.381	0.790	0.575	0.787			16
	GLY	A	17	0.831	0.998							17
	PRO	A	18	1.000	0.980	1.000	1.000				18	18
	GLY	A	19	0.974	0.905						19	19
	CYS	A	20	0.881	0.993	0.930						20
	CYS	A	21	0.989	0.930	0.887					21	21
	ASN	A	22	0.915	0.983	0.054	0.521				22	22
	GLY	A	23	0.971	0.750							
	LYS	A	24	0.786	0.989	0.367	0.850	0.518	0.366			
	CYS	A	25	0.988	0.983	0.925					25	25
	ILE	A	26	0.968	0.991	0.632	0.372				26	26
	CYS	A	27	0.997	0.989	0.920					27	27
	ARG	A	28	0.979	0.992	0.460	0.696	0.747	0.391	1.000	28	28
	THR	A	29	0.901	0.976	0.697					29	29
	VAL	A	30	0.898	0.978	0.327						30
	PRO	A	31	1.000	0.993	1.000	1.000				31	31
	ARG	A	32	0.999	0.986	0.641	0.691	0.438	0.462	1.000	32	32
	LYS	A	33	0.996	0.992	0.526	0.949	0.627	0.367		33	33
	GLY	A	34	0.984	0.984						34	34
	VAL	A	35	0.999	0.989	0.233					35	35
	ASN	A	36	0.844	0.998	0.769	0.275					36
	SER	A	37	0.990	0.989	0.118					37	37
	CYS	A	38	0.998	0.995	0.991					38	38
	ARG	A	39	0.995	0.981	0.678	0.167	0.480	0.675	1.000	39	39
	CYS	A	40	0.992	0.984	0.803					40	40
	MET	A	41	0.926		0.894	0.684	0.716				

JPEG image of S(phi)~Residue_number Plot

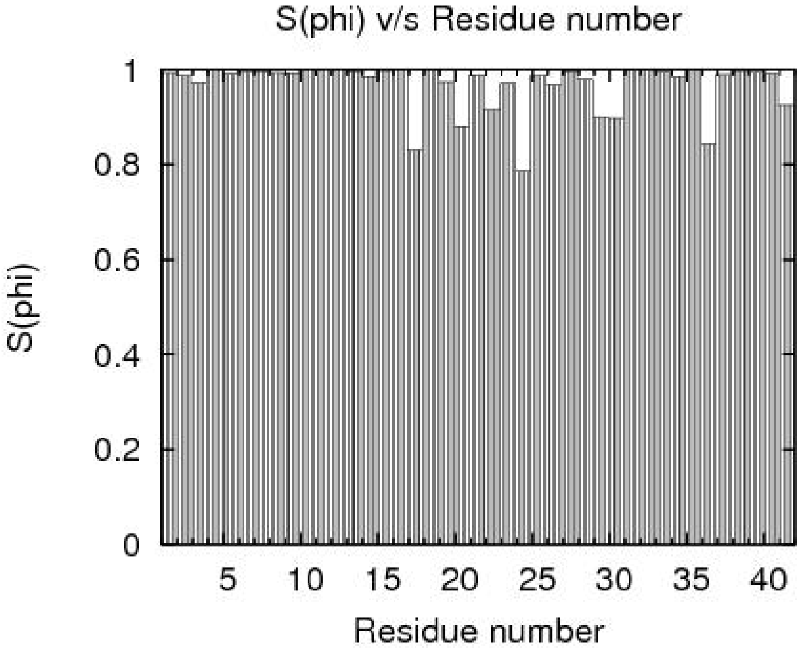

JPEG image of S(psi)~Residue_number Plot

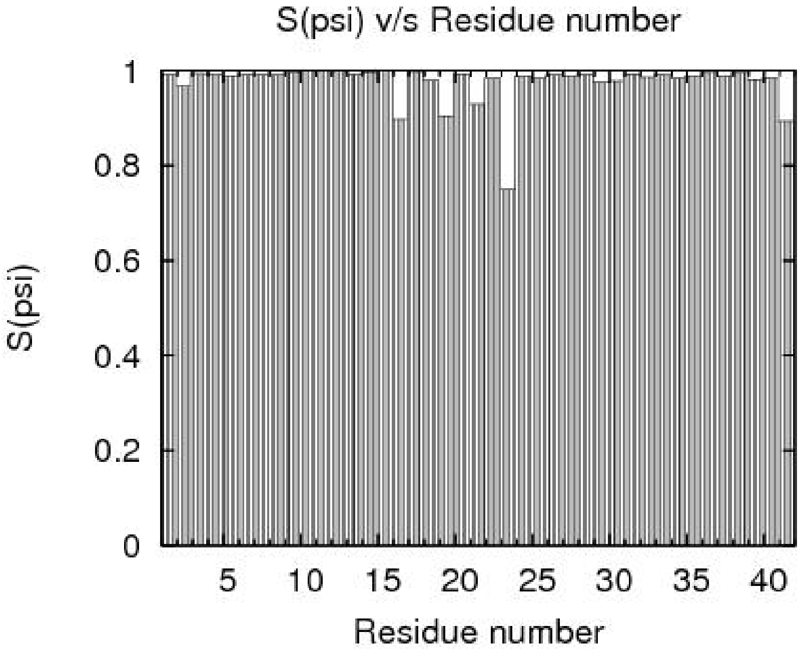

Table of Backbone and Heavy Atom RMSD

Text report of backbone and heavy atom RMSD for ordered regions

```
>
> Kabsch RMSD data for family `TboIT1.pdb'
>
> Kabsch RMSD of backbone atoms in res. A[2..15],A[18..19],A[21..22],A[25..29],A[31..35],A[37..4
> Kabsch RMSD of backbone atoms in res. A[2..15],A[18..19],A[21..22],A[25..29],A[31..35],A[37..4
```

```
> Kabsch RMSD of backbone atoms in res. A[2..15],A[18..19],A[21..22],A[25..29],A[31..35],A[37..4
> Kabsch RMSD of backbone atoms in res. A[2..15],A[18..19],A[21..22],A[25..29],A[31..35],A[37..4
> Kabsch RMSD of backbone atoms in res. A[2..15],A[18..19],A[21..22],A[25..29],A[31..35],A[37..4
> Kabsch RMSD of backbone atoms in res. A[2..15],A[18..19],A[21..22],A[25..29],A[31..35],A[37..4
> Kabsch RMSD of backbone atoms in res. A[2..15],A[18..19],A[21..22],A[25..29],A[31..35],A[37..4
> Kabsch RMSD of backbone atoms in res. A[2..15],A[18..19],A[21..22],A[25..29],A[31..35],A[37..4
> Kabsch RMSD of backbone atoms in res. A[2..15],A[18..19],A[21..22],A[25..29],A[31..35],A[37..4
> Kabsch RMSD of backbone atoms in res. A[2..15],A[18..19],A[21..22],A[25..29],A[31..35],A[37..4
> Kabsch RMSD of backbone atoms in res. A[2..15],A[18..19],A[21..22],A[25..29],A[31..35],A[37..4
> Kabsch RMSD of backbone atoms in res. A[2..15],A[18..19],A[21..22],A[25..29],A[31..35],A[37..4
> Kabsch RMSD of backbone atoms in res. A[2..15],A[18..19],A[21..22],A[25..29],A[31..35],A[37..4
> Kabsch RMSD of backbone atoms in res. A[2..15],A[18..19],A[21..22],A[25..29],A[31..35],A[37..4
> Kabsch RMSD of backbone atoms in res. A[2..15],A[18..19],A[21..22],A[25..29],A[31..35],A[37..4
> Kabsch RMSD of backbone atoms in res. A[2..15],A[18..19],A[21..22],A[25..29],A[31..35],A[37..4
> Kabsch RMSD of backbone atoms in res. A[2..15],A[18..19],A[21..22],A[25..29],A[31..35],A[37..4
> Kabsch RMSD of backbone atoms in res. A[2..15],A[18..19],A[21..22],A[25..29],A[31..35],A[37..4
> Kabsch RMSD of backbone atoms in res. A[2..15],A[18..19],A[21..22],A[25..29],A[31..35],A[37..4
> Kabsch RMSD of backbone atoms in res. A[2..15],A[18..19],A[21..22],A[25..29],A[31..35],A[37..4
>
> Kabsch RMSD statistics for 20 structures:
> Mean RMSD using as refer. str. `average' for res.[2..15],[18..19],[21..22],[25..29],[31..35],[
> Range of RMSD values to reference struct. is 0.297 to 1.067


> Kabsch RMSD of heavy atoms in res. A[2..15],A[18..19],A[21..22],A[25..29],A[31..35],A[37..40],
> Kabsch RMSD of heavy atoms in res. A[2..15],A[18..19],A[21..22],A[25..29],A[31..35],A[37..40],
> Kabsch RMSD of heavy atoms in res. A[2..15],A[18..19],A[21..22],A[25..29],A[31..35],A[37..40],
> Kabsch RMSD of heavy atoms in res. A[2..15],A[18..19],A[21..22],A[25..29],A[31..35],A[37..40],
> Kabsch RMSD of heavy atoms in res. A[2..15],A[18..19],A[21..22],A[25..29],A[31..35],A[37..40],
> Kabsch RMSD of heavy atoms in res. A[2..15],A[18..19],A[21..22],A[25..29],A[31..35],A[37..40],
> Kabsch RMSD of heavy atoms in res. A[2..15],A[18..19],A[21..22],A[25..29],A[31..35],A[37..40],
> Kabsch RMSD of heavy atoms in res. A[2..15],A[18..19],A[21..22],A[25..29],A[31..35],A[37..40],
> Kabsch RMSD of heavy atoms in res. A[2..15],A[18..19],A[21..22],A[25..29],A[31..35],A[37..40],
> Kabsch RMSD of heavy atoms in res. A[2..15],A[18..19],A[21..22],A[25..29],A[31..35],A[37..40],
> Kabsch RMSD of heavy atoms in res. A[2..15],A[18..19],A[21..22],A[25..29],A[31..35],A[37..40],
> Kabsch RMSD of heavy atoms in res. A[2..15],A[18..19],A[21..22],A[25..29],A[31..35],A[37..40],
> Kabsch RMSD of heavy atoms in res. A[2..15],A[18..19],A[21..22],A[25..29],A[31..35],A[37..40],
> Kabsch RMSD of heavy atoms in res. A[2..15],A[18..19],A[21..22],A[25..29],A[31..35],A[37..40],
> Kabsch RMSD of heavy atoms in res. A[2..15],A[18..19],A[21..22],A[25..29],A[31..35],A[37..40],
> Kabsch RMSD of heavy atoms in res. A[2..15],A[18..19],A[21..22],A[25..29],A[31..35],A[37..40],
> Kabsch RMSD of heavy atoms in res. A[2..15],A[18..19],A[21..22],A[25..29],A[31..35],A[37..40],
> Kabsch RMSD of heavy atoms in res. A[2..15],A[18..19],A[21..22],A[25..29],A[31..35],A[37..40],
> Kabsch RMSD of heavy atoms in res. A[2..15],A[18..19],A[21..22],A[25..29],A[31..35],A[37..40],
> Kabsch RMSD of heavy atoms in res. A[2..15],A[18..19],A[21..22],A[25..29],A[31..35],A[37..40],
>
> Kabsch RMSD statistics for 20 structures:
> Mean RMSD using as refer. str. `average' for res.[2..15],[18..19],[21..22],[25..29],[31..35],[
> Range of RMSD values to reference struct. is 0.989 to 2.171
```

Text report of backbone RMSD for entire protein

```
> Kabsch RMSD of backb atoms in res. *[1..41],for model  1 is: 0.462
> Kabsch RMSD of backb atoms in res. *[1..41],for model  2 is: 0.356
> Kabsch RMSD of backb atoms in res. *[1..41],for model  3 is: 0.661
> Kabsch RMSD of backb atoms in res. *[1..41],for model  4 is: 0.652
> Kabsch RMSD of backb atoms in res. *[1..41],for model  5 is: 0.534
> Kabsch RMSD of backb atoms in res. *[1..41],for model  6 is: 0.575
> Kabsch RMSD of backb atoms in res. *[1..41],for model  7 is: 0.644
> Kabsch RMSD of backb atoms in res. *[1..41],for model  8 is: 0.348 (*)
> Kabsch RMSD of backb atoms in res. *[1..41],for model  9 is: 0.551
> Kabsch RMSD of backb atoms in res. *[1..41],for model 10 is: 0.539
> Kabsch RMSD of backb atoms in res. *[1..41],for model 11 is: 0.386
```

```
> Kabsch RMSD of backb atoms in res. *[1..41],for model 12 is: 0.742
> Kabsch RMSD of backb atoms in res. *[1..41],for model 13 is: 0.492
> Kabsch RMSD of backb atoms in res. *[1..41],for model 14 is: 0.562
> Kabsch RMSD of backb atoms in res. *[1..41],for model 15 is: 0.448
> Kabsch RMSD of backb atoms in res. *[1..41],for model 16 is: 1.074
> Kabsch RMSD of backb atoms in res. *[1..41],for model 17 is: 0.382
> Kabsch RMSD of backb atoms in res. *[1..41],for model 18 is: 0.428
> Kabsch RMSD of backb atoms in res. *[1..41],for model 19 is: 1.042
> Kabsch RMSD of backb atoms in res. *[1..41],for model 20 is: 0.498
>
> Kabsch RMSD statistics for 20 structures:
> Mean RMSD using as refer. str. `average' for res.[1..41], is: 0.569
> Range of RMSD values to reference struct. is 0.348 to 1.074
```

Text report of heavy atom RMSD for entire protein

```
> Kabsch RMSD of heavy atoms in res. *[1..41],for model  1 is: 1.155
> Kabsch RMSD of heavy atoms in res. *[1..41],for model  2 is: 1.089
> Kabsch RMSD of heavy atoms in res. *[1..41],for model  3 is: 1.327
> Kabsch RMSD of heavy atoms in res. *[1..41],for model  4 is: 1.224
> Kabsch RMSD of heavy atoms in res. *[1..41],for model  5 is: 1.263
> Kabsch RMSD of heavy atoms in res. *[1..41],for model  6 is: 1.228
> Kabsch RMSD of heavy atoms in res. *[1..41],for model  7 is: 1.220
> Kabsch RMSD of heavy atoms in res. *[1..41],for model  8 is: 1.093
> Kabsch RMSD of heavy atoms in res. *[1..41],for model  9 is: 1.270
> Kabsch RMSD of heavy atoms in res. *[1..41],for model 10 is: 1.282
> Kabsch RMSD of heavy atoms in res. *[1..41],for model 11 is: 1.048
> Kabsch RMSD of heavy atoms in res. *[1..41],for model 12 is: 1.526
> Kabsch RMSD of heavy atoms in res. *[1..41],for model 13 is: 1.154
> Kabsch RMSD of heavy atoms in res. *[1..41],for model 14 is: 1.225
> Kabsch RMSD of heavy atoms in res. *[1..41],for model 15 is: 1.083
> Kabsch RMSD of heavy atoms in res. *[1..41],for model 16 is: 2.059
> Kabsch RMSD of heavy atoms in res. *[1..41],for model 17 is: 1.287
> Kabsch RMSD of heavy atoms in res. *[1..41],for model 18 is: 1.018 (*)
> Kabsch RMSD of heavy atoms in res. *[1..41],for model 19 is: 1.950
> Kabsch RMSD of heavy atoms in res. *[1..41],for model 20 is: 1.149
>
> Kabsch RMSD statistics for 20 structures:
> Mean RMSD using as refer. str. `average' for res.[1..41], is: 1.282
> Range of RMSD values to reference struct. is 1.018 to 2.059
```

Summary of heavy atom and backbone RMSDs over the whole protein and ordered residues

```
RMSD Values
        all residues    ordered residues        selected residues
All backbone atoms      0.6     0.6     0.6
All heavy atoms 1.3     1.3     1.3
```

Output from PROCHECK

Ramachandran Plot for all models

Text summary of Ramachandran Plot

```
+----------<<< P  R  O  C  H  E  C  K     S  U  M  M  A  R  Y  >>>----------+
|                                                                           |
| TboIT1_020.rin   0.0                                         820 residues |
```

```
 |                                                                             |
*| Ramachandran plot:   67.3% core   31.9% allow    0.8% gener    0.0% disall |
 |                                                                             |
*| All Ramachandrans:   47 labelled residues (out of 780)                      |
+| Chi1-chi2 plots:      6 labelled residues (out of 500)                      |
```

JPEG image for all model Ramachandran Plot

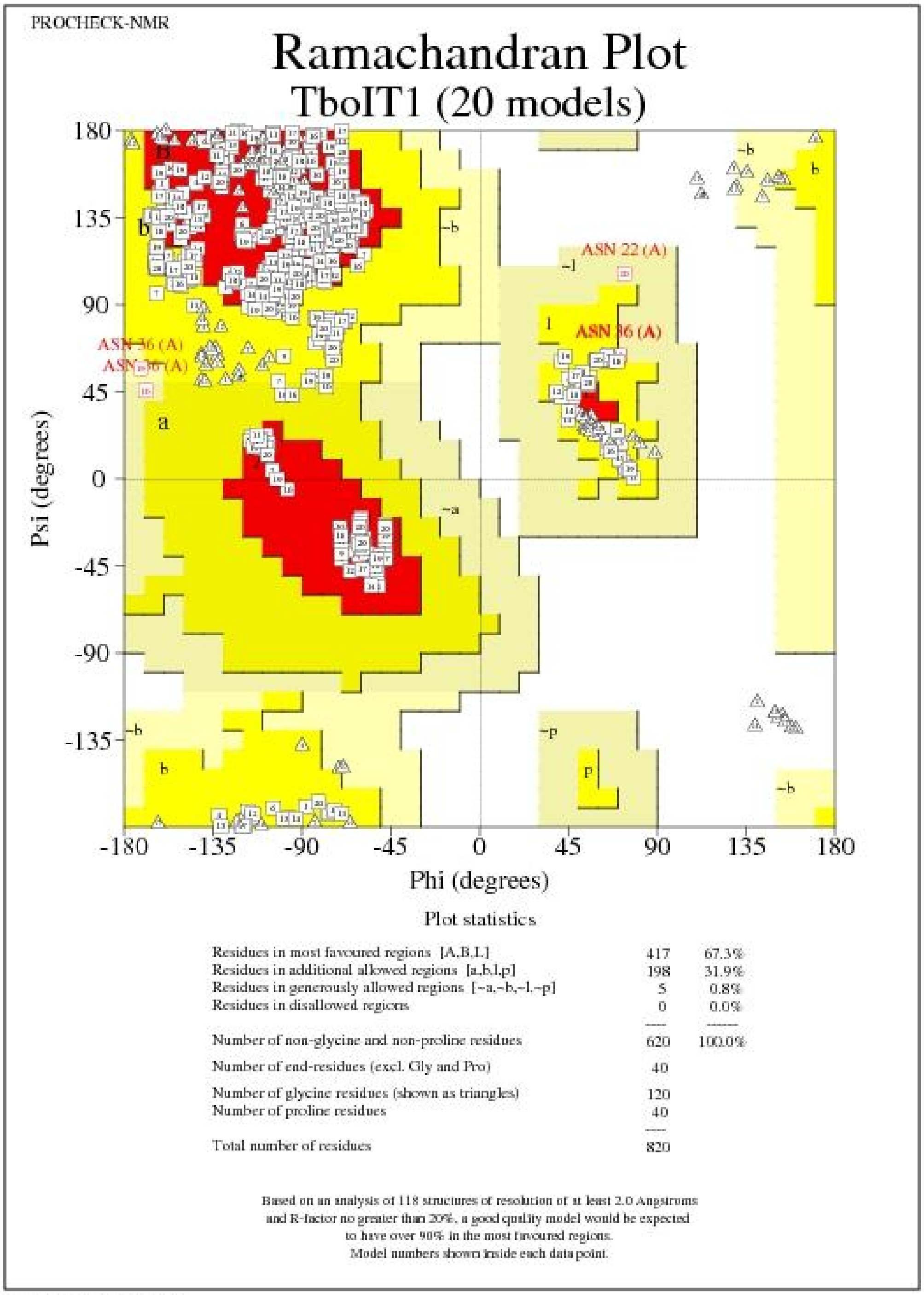

TboIT1_01_ramachand.ps

Residue Properties for all models

JPEG for all model Residue Properties

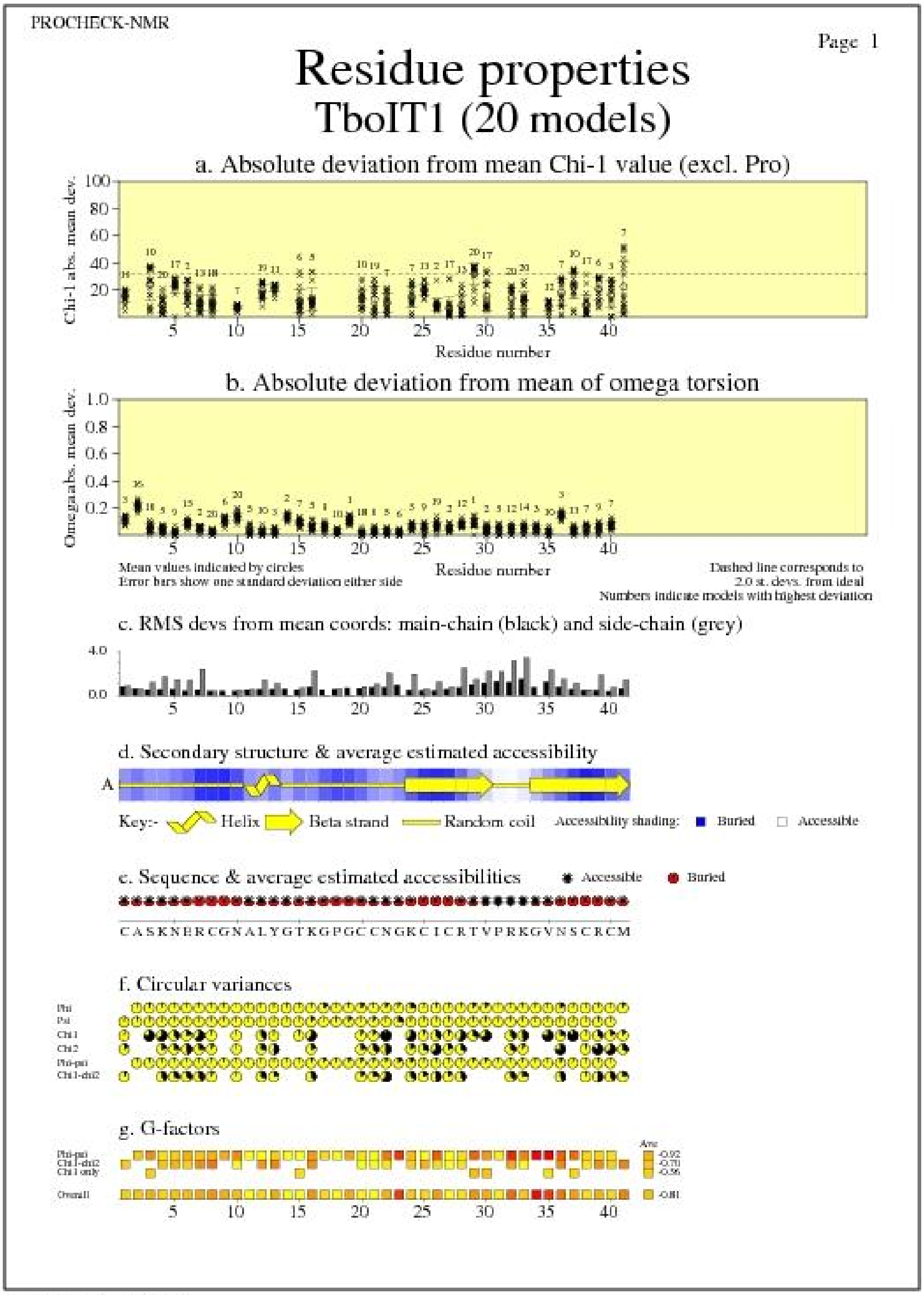

TboIT1_10_residprop.ps

Model Secondary Structures from Procheck

JPEG for Model Secondary Structures - page $num_n

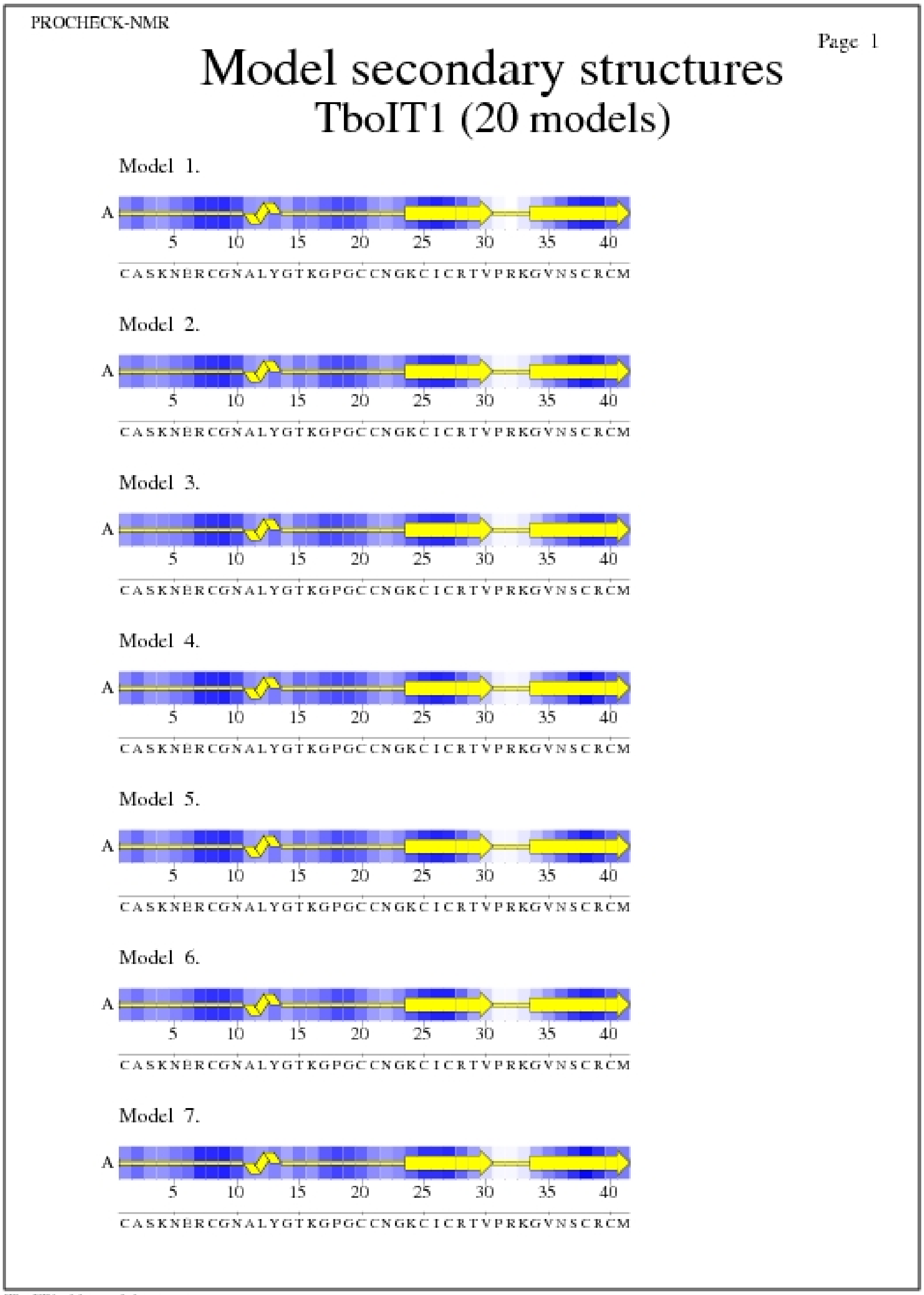

TboIT1_11_modelsecs.ps

JPEG for Model Secondary Structures - page $num_n

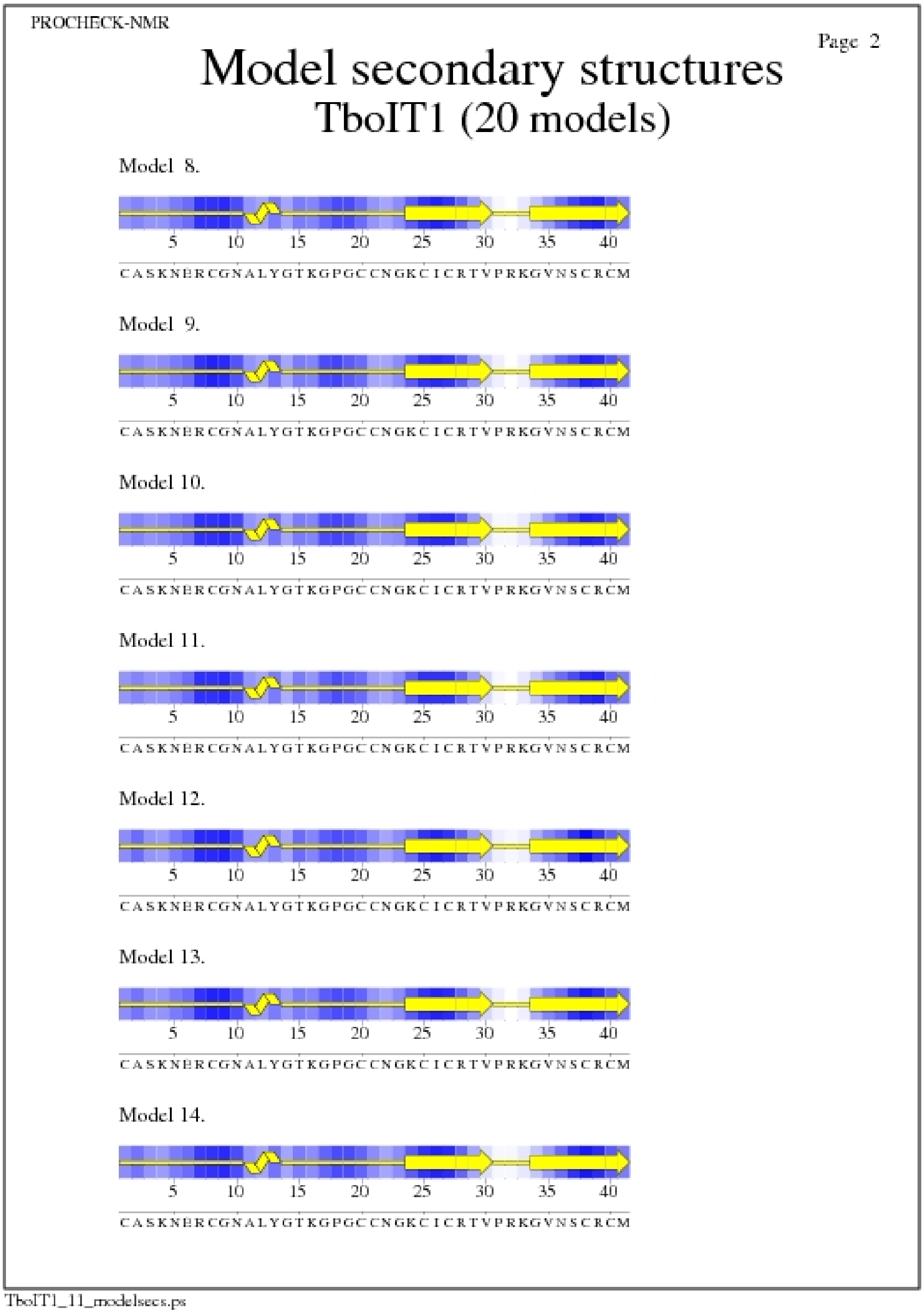

JPEG for Model Secondary Structures - page $num_n

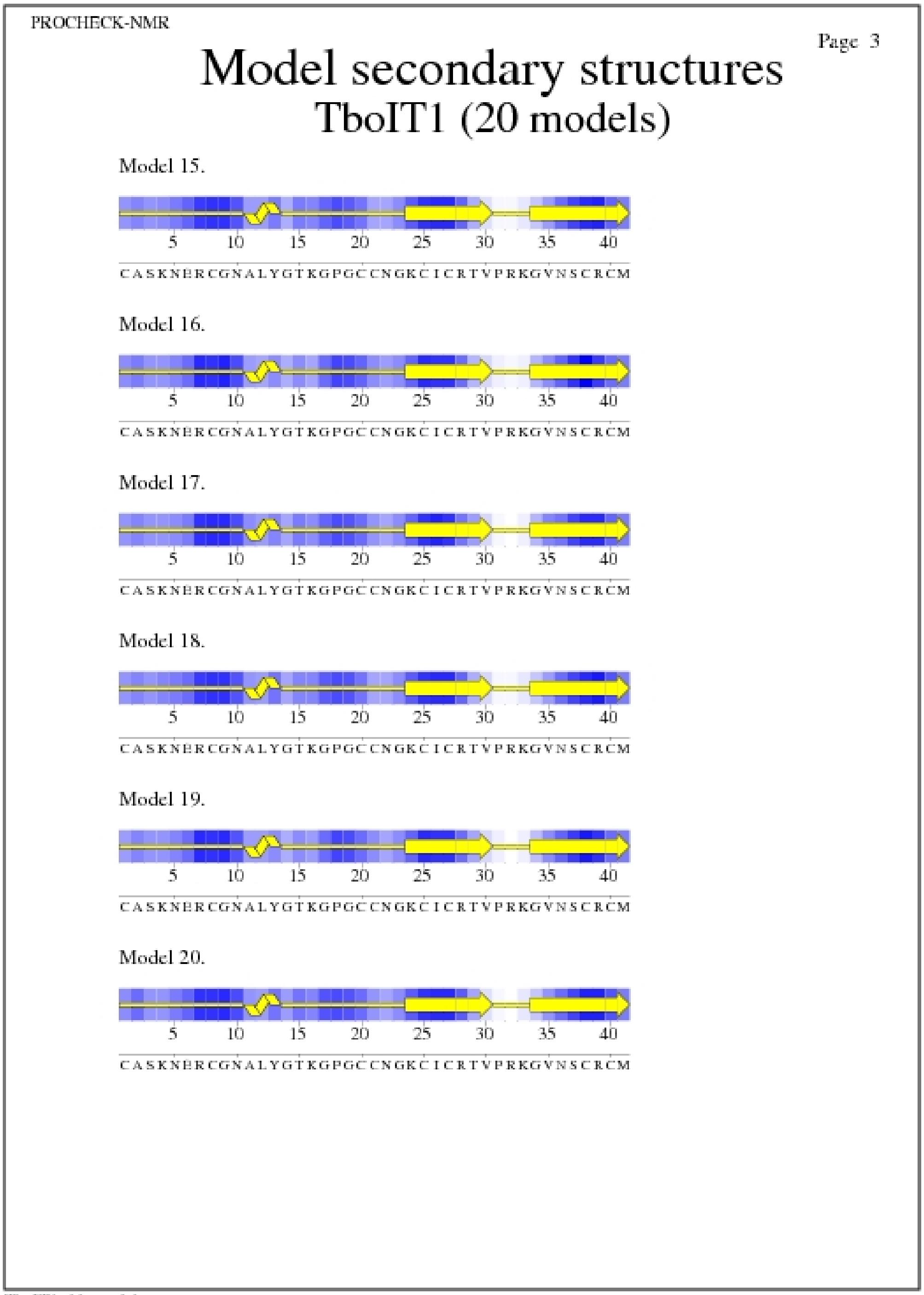

TboIT1_11_modelsecs.ps

Ramachandran Plots for each residue

JPEG for residue Ramachandran Plots - page $num_n

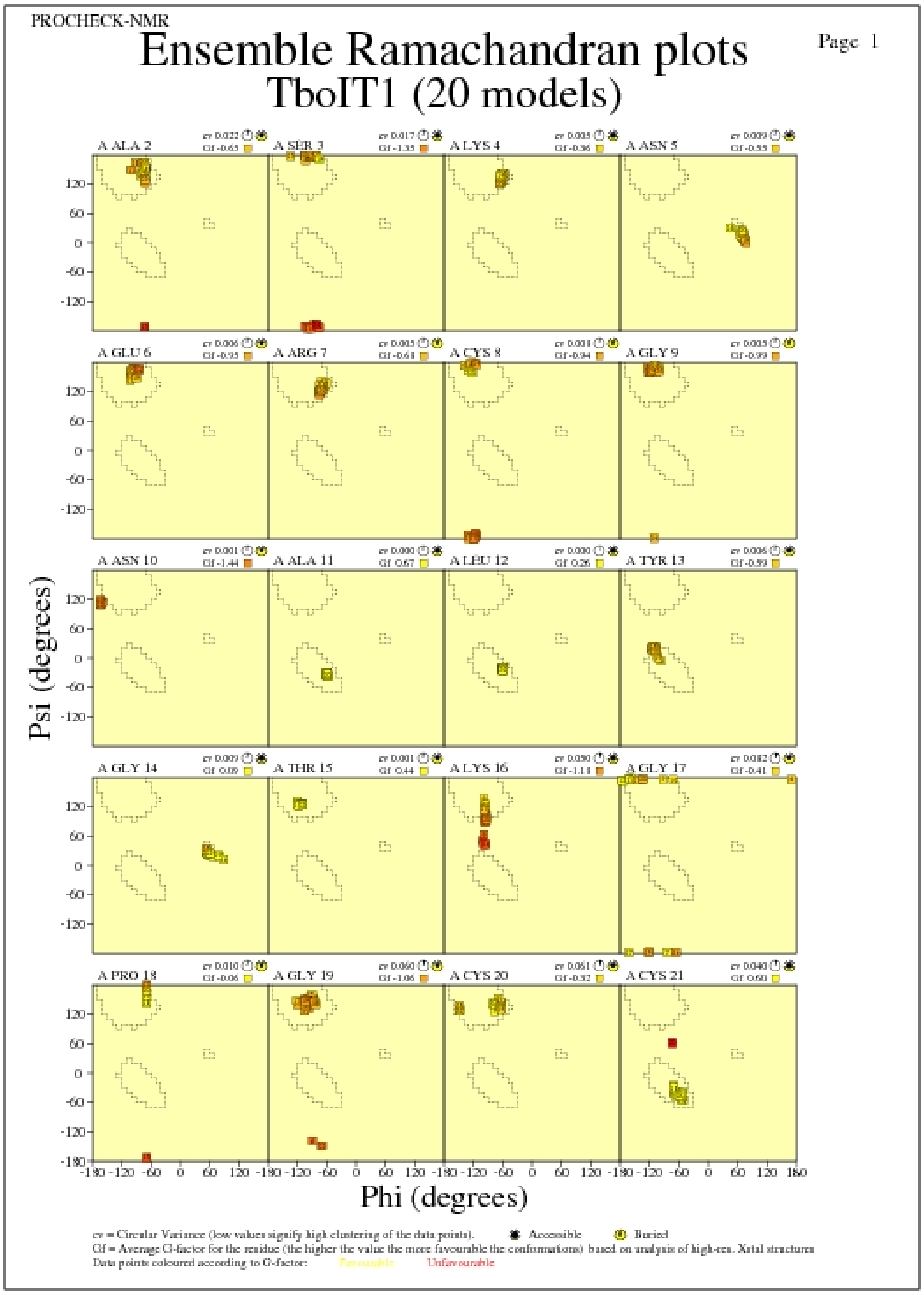

TboIT1_08_ensramach.ps

JPEG for residue Ramachandran Plots - page $num_n

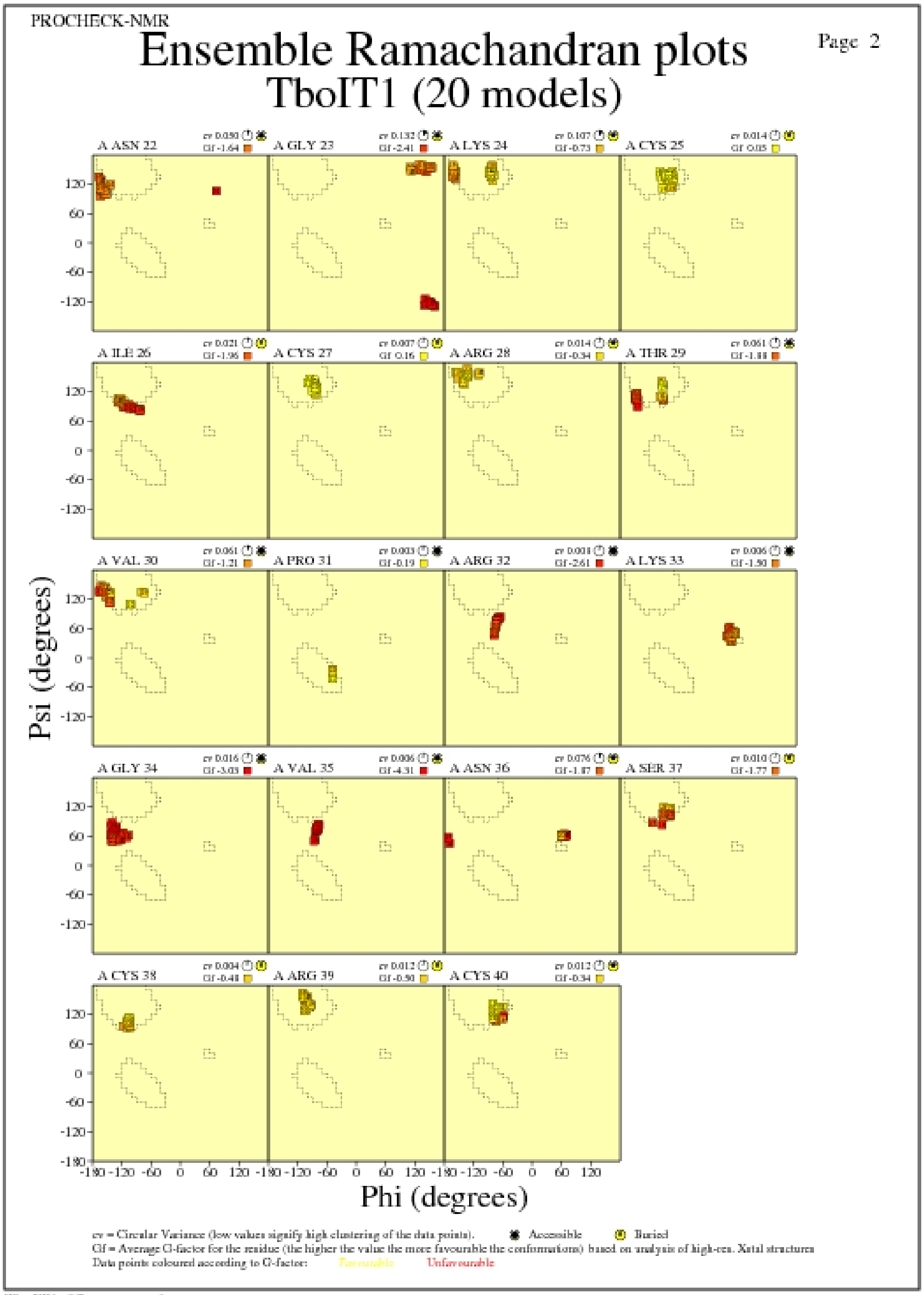

TboIT1_08_ensramach.ps

Ramachandran analysis for each residue from Molprobity

Chi1-Chi2 Plots for each residue

JPEG for residue Chi1-Chi2 Plots - page $num_n

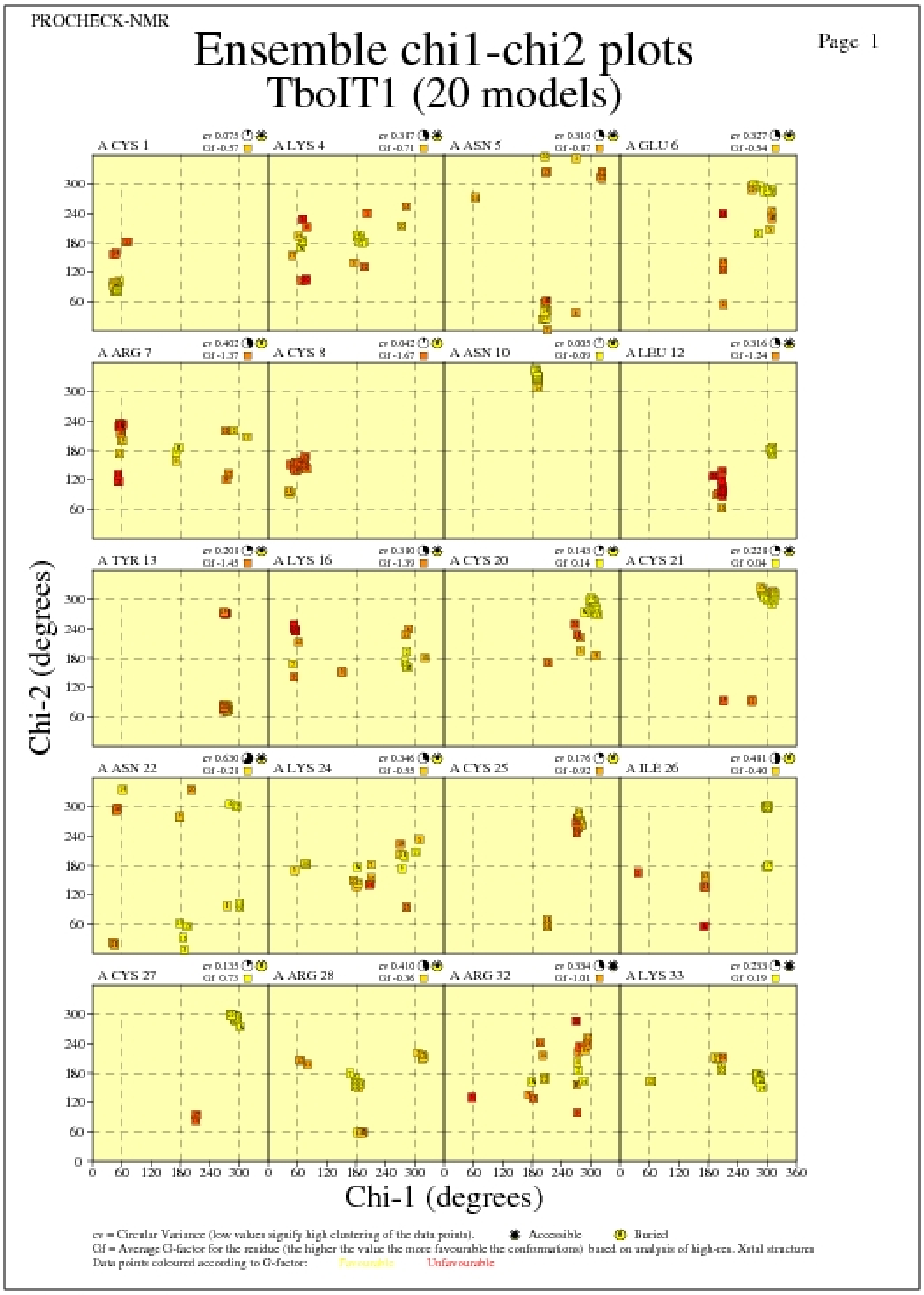

TboIT1_09_ensch1ch2.ps

JPEG for residue Chi1-Chi2 Plots - page $num_n

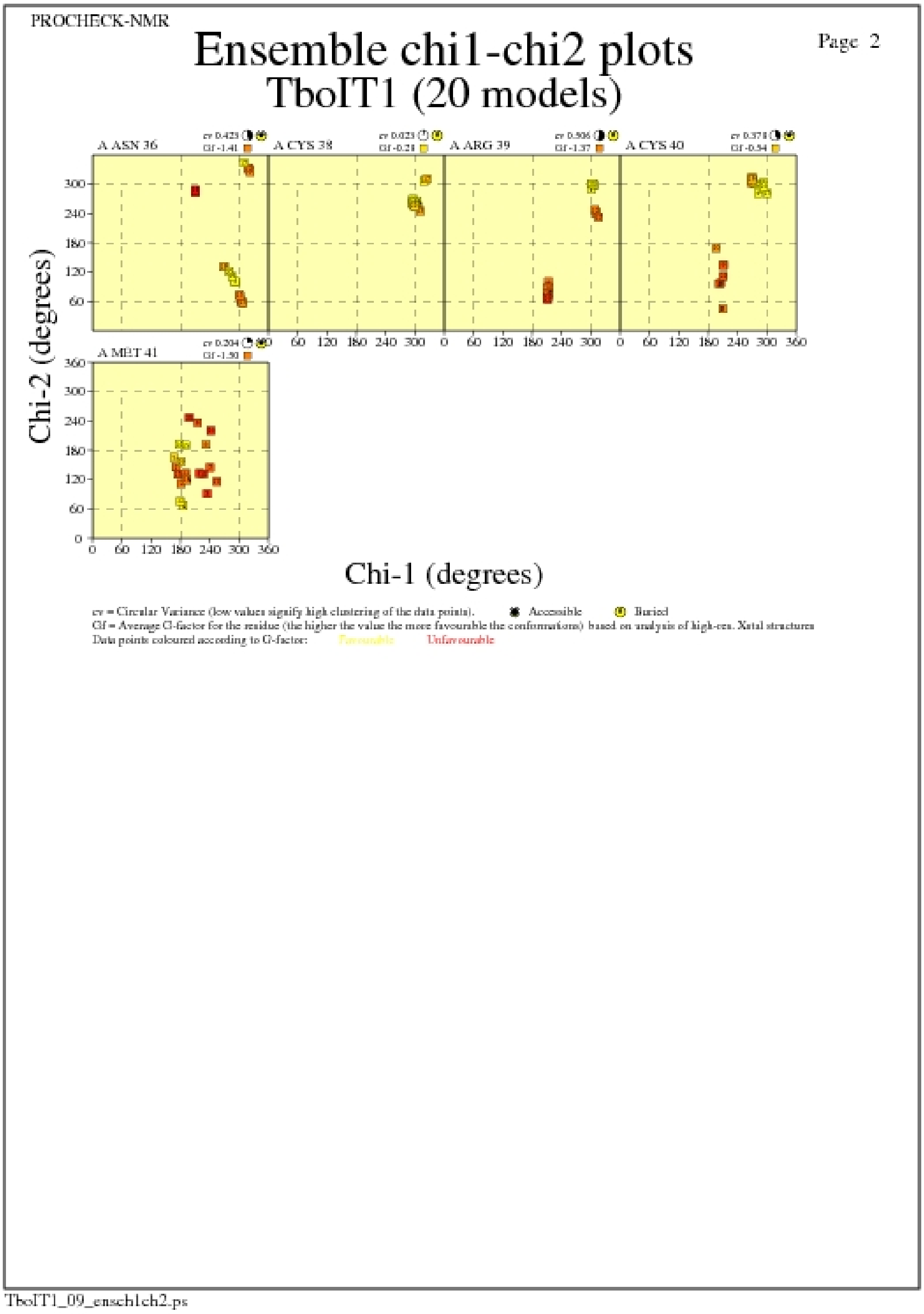

Procheck G-factors for phi-psi for each residue

JPEG image for residue phi-psi G-factors

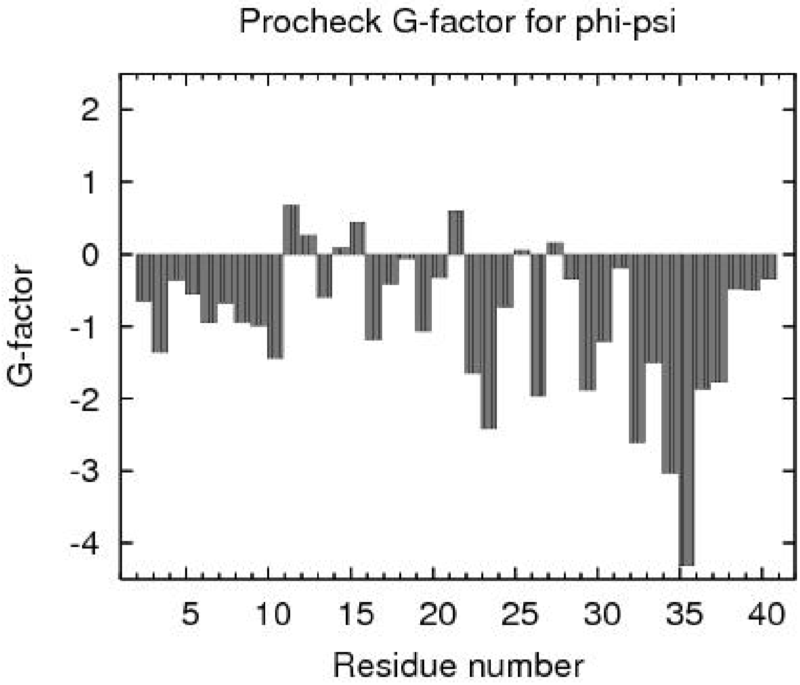

Table of Procheck G-factors for phi-psi for ordered residues

```
No ordered residues found for phipsi_gfactor.
```

Procheck G-factors for all dihedral angles for each residue

JPEG image for residue all dihedral G-factors

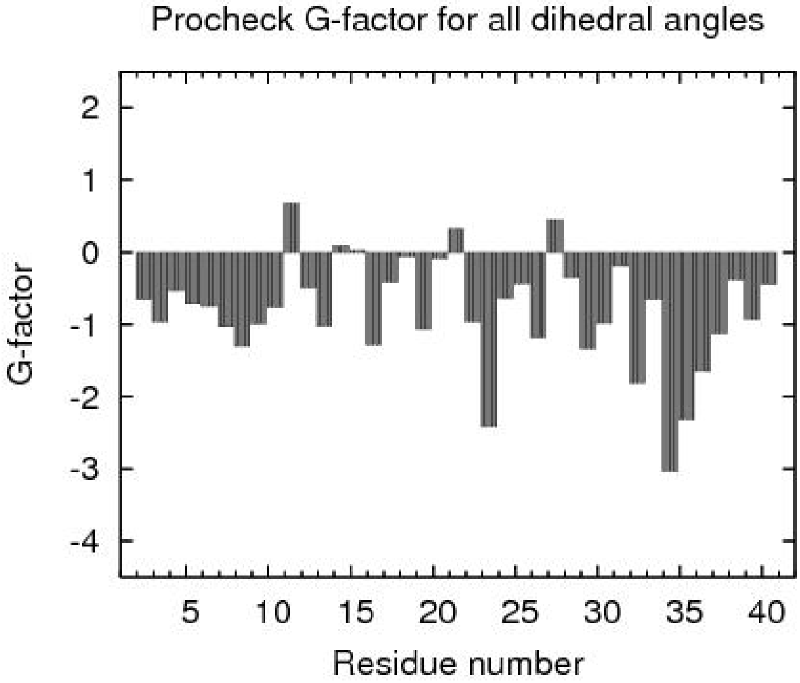

Table of Procheck G-factors for all dihedrals for ordered residues

```
No ordered residues found for alldih_gfactor.
```

Output from Verify3D

Verify3D Score over a window of $winsize_s residues

JPEG image for Verify3D Score

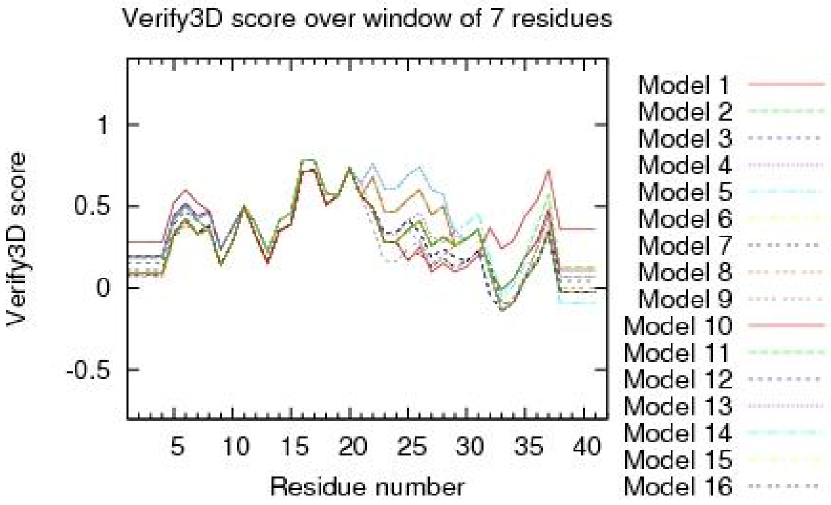

Table of Verify3D scores for ordered residues across all models

```
No ordered residues found for verify3d.
```

Output from Prosall

Prosall Score over a window of $winsize_s residues

JPEG image for Prosall Score

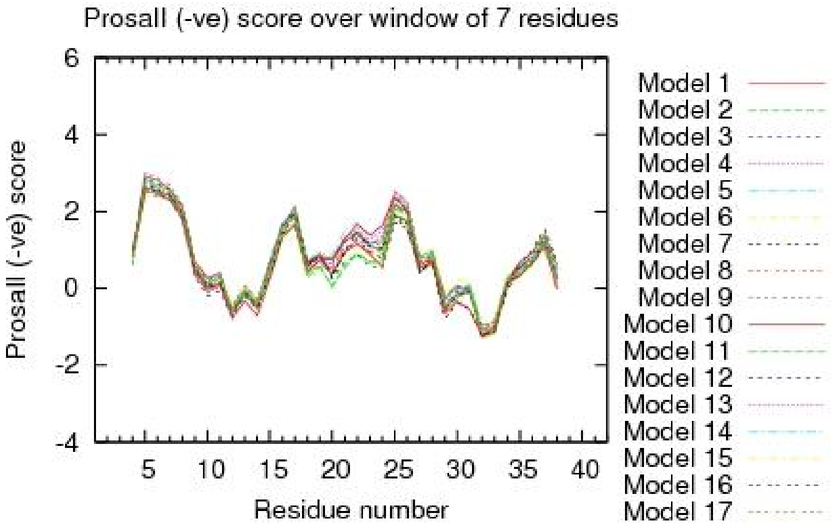

Table of Verify3D scores for ordered residues across all models

```
No ordered residues found for verify3d.
```

Output from MolProbity

VdW violations from MAGE

JPEG image for MAGE VdW violation

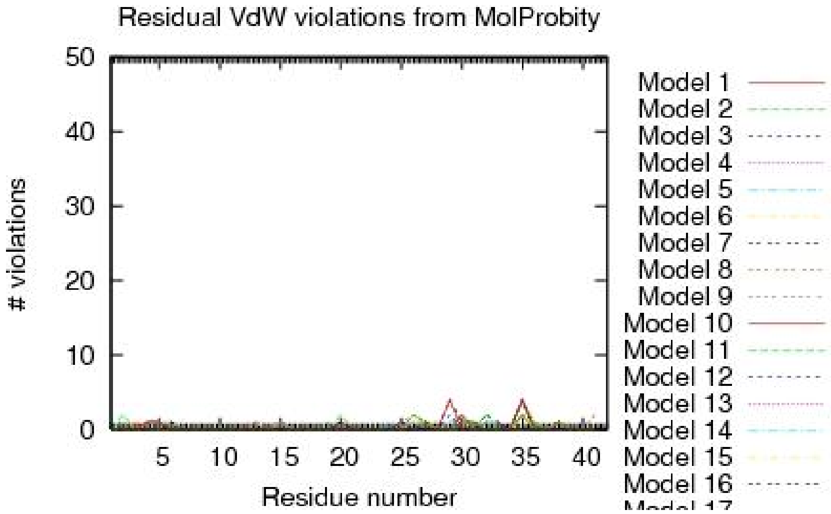

Table of MAGE VdW violations for ordered residues across all models

```
No ordered residues found for mage_clash.
```

List of bad contacts calculated by MAGE for model $num_n

```
/farm/software/bin/probe

:   585:A  25 CYS  SG  :A  20 CYS  SG  :   -0.854:       74
```

```
:   585:A  13 TYR  O   :A  15 THR 3HG2 :   -0.624:       71

:   585:A  35 VAL 3HG1 :A  35 VAL  O   :   -0.505:       33
:   585:A  35 VAL  O   :A  35 VAL 2HG2 :   -0.485:       33

:   585:A  31 PRO 1HD  :A  30 VAL  CG2 :   -0.481:       61

:   585:A  27 CYS  HA  :A  38 CYS  HA  :   -0.463:       43

:   585:A   3 SER  O   :A   6 GLU 1HB  :   -0.405:       44
#sum2 ::11.97 clashscore : 6.94 clashscore B
```

List of bad contacts calculated by MAGE for model $num_n

```
/farm/software/bin/probe

:   585:A  25 CYS  SG  :A  20 CYS  SG  :   -0.659:       51
:   585:A   2 ALA 1HB  :A   6 GLU 2HB  :   -0.461:       23
:   585:A   2 ALA 3HB  :A  20 CYS  HA  :   -0.421:       53

:   585:A  15 THR 1HG2 :A  36 ASN  OD1 :   -0.496:       60

:   585:A  35 VAL 2HG1 :A  35 VAL  O   :   -0.489:       64

:   585:A  27 CYS  HA  :A  38 CYS  HA  :   -0.451:       70
#sum2 ::10.26 clashscore : 3.36 clashscore B
```

List of bad contacts calculated by MAGE for model $num_n

```
/farm/software/bin/probe

:   585:A  13 TYR  O   :A  15 THR 3HG2 :   -0.628:       31

:   585:A  35 VAL 2HG1 :A  35 VAL  O   :   -0.460:       40

:   585:A  27 CYS  HA  :A  38 CYS  HA  :   -0.458:       71

:   585:A   4 LYS  O   :A   5 ASN  CB  :   -0.415:       26
#sum2 ::6.84 clashscore : 6.71 clashscore B
```

List of bad contacts calculated by MAGE for model $num_n

```
/farm/software/bin/probe

:   585:A  25 CYS  SG  :A  20 CYS  SG  :   -0.688:       53

:   585:A  13 TYR  O   :A  15 THR 3HG2 :   -0.536:       52

:   585:A  30 VAL  O   :A  30 VAL 2HG1 :   -0.406:       23

:   585:A   3 SER  O   :A   6 GLU 1HB  :   -0.401:       72
#sum2 ::6.84 clashscore : 3.48 clashscore B
```

List of bad contacts calculated by MAGE for model $num_n

```
/farm/software/bin/probe

:   585:A  30 VAL 3HG1 :A  29 THR  O   :   -0.536:       72
:   585:A  32 ARG 2HG  :A  30 VAL 3HG2 :   -0.462:       55

:   585:A  35 VAL 3HG1 :A  35 VAL  O   :   -0.510:       45
:   585:A  35 VAL  O   :A  35 VAL 2HG2 :   -0.495:       70

:   585:A  27 CYS  HA  :A  38 CYS  HA  :   -0.484:       61
#sum2 ::8.55 clashscore : 0.00 clashscore B
```

List of bad contacts calculated by MAGE for model $num_n

```
/farm/software/bin/probe

:   585:A  27 CYS  HA  :A  38 CYS  HA  :   -0.447:       23

:   585:A  30 VAL 3HG1 :A  29 THR  O   :   -0.429:       72

:   585:A   3 SER  O   :A   6 GLU 1HB  :   -0.427:       64
#sum2 ::5.13 clashscore : 3.77 clashscore B
```

List of bad contacts calculated by MAGE for model $num_n

```
/farm/software/bin/probe

:   585:A   2 ALA 1HB  :A   6 GLU 2HB  :   -0.567:       41

:   585:A  35 VAL 3HG1 :A  35 VAL  O   :   -0.502:       33
:   585:A  35 VAL  O   :A  35 VAL 2HG2 :   -0.445:       71

:   585:A  25 CYS  SG  :A  20 CYS  SG  :   -0.470:       65

:   585:A  30 VAL 2HG1 :A  32 ARG  H   :   -0.458:       32
:   585:A  32 ARG  O   :A  33 LYS  C   :   -0.443:       61

:   585:A  27 CYS  HA  :A  38 CYS  HA  :   -0.404:       40
#sum2 ::11.97 clashscore : 6.76 clashscore B
```

List of bad contacts calculated by MAGE for model $num_n

```
/farm/software/bin/probe

:   585:A  15 THR 1HG2 :A  36 ASN  OD1 :   -0.501:       20

:   585:A  27 CYS  HA  :A  38 CYS  HA  :   -0.488:       34

:   585:A  35 VAL 2HG1 :A  35 VAL  O   :   -0.485:       22

:   585:A  26 ILE  O   :A  26 ILE 3HG2 :   -0.449:       63

:   585:A   4 LYS  O   :A   5 ASN  CB  :   -0.435:       52

:   585:A  25 CYS  SG  :A  20 CYS  SG  :   -0.423:       71
```

```
:   585:A  32 ARG  O   :A  33 LYS  CG  :   -0.402:       42
#sum2 ::11.97 clashscore : 10.49 clashscore B
```

List of bad contacts calculated by MAGE for model $num_n

```
/farm/software/bin/probe

:   585:A  13 TYR  O   :A  15 THR 3HG2 :   -0.533:       60

:   585:A  35 VAL 3HG1 :A  35 VAL  O   :   -0.505:       55
:   585:A  35 VAL  O   :A  35 VAL 2HG2 :   -0.420:       55

:   585:A  27 CYS  HA  :A  38 CYS  HA  :   -0.446:       72

:   585:A  30 VAL  O   :A  30 VAL 2HG1 :   -0.437:       40

:   585:A  28 ARG 2HG  :A  26 ILE 2HG2 :   -0.400:       61
#sum2 ::10.26 clashscore : 0.00 clashscore B
```

List of bad contacts calculated by MAGE for model $num_n

```
/farm/software/bin/probe

:   585:A  27 CYS  HA  :A  38 CYS  HA  :   -0.475:       61

:   585:A  31 PRO 1HD  :A  30 VAL  CG2 :   -0.463:       34

:   585:A  35 VAL 2HG1 :A  35 VAL  O   :   -0.421:       62

:   585:A   4 LYS  O   :A   5 ASN  CB  :   -0.410:       44
#sum2 ::6.84 clashscore : 3.58 clashscore B
```

List of bad contacts calculated by MAGE for model $num_n

```
/farm/software/bin/probe

:   585:A  15 THR 1HG2 :A  36 ASN  OD1 :   -0.536:       34

:   585:A  35 VAL 3HG1 :A  35 VAL  O   :   -0.500:       42
:   585:A  35 VAL  O   :A  35 VAL 2HG2 :   -0.454:       54

:   585:A  27 CYS  HA  :A  38 CYS  HA  :   -0.471:       72

:   585:A  26 ILE  O   :A  26 ILE 3HG2 :   -0.460:       55

:   585:A  30 VAL 2HG1 :A  32 ARG  H   :   -0.451:       53
:   585:A  32 ARG  N   :A  30 VAL 2HG1 :   -0.415:       53

:   585:A   4 LYS  O   :A   5 ASN  CB  :   -0.402:       60
#sum2 ::13.68 clashscore : 3.40 clashscore B
```

List of bad contacts calculated by MAGE for model $num_n

```
/farm/software/bin/probe

:   585:A  25 CYS  SG  :A  20 CYS  SG  :   -0.570:       72
```

```
:   585:A  13 TYR  O   :A  15 THR 3HG2 :   -0.533:       71

:   585:A  35 VAL 3HG1 :A  35 VAL  O   :   -0.495:       73
:   585:A  35 VAL  O   :A  35 VAL 2HG2 :   -0.413:       73

:   585:A  29 THR 3HG2 :A  29 THR  O   :   -0.424:       72

:   585:A  32 ARG  O   :A  33 LYS  C   :   -0.411:       21

:   585:A  27 CYS  HA  :A  38 CYS  HA  :   -0.402:       64
#sum2 ::11.97 clashscore : 3.23 clashscore B
```

List of bad contacts calculated by MAGE for model $num_n

```
/farm/software/bin/probe

:   585:A  13 TYR  O   :A  15 THR 3HG2 :   -0.556:       44

:   585:A  35 VAL 3HG1 :A  35 VAL  O   :   -0.497:       71
:   585:A  35 VAL  O   :A  35 VAL 2HG2 :   -0.438:       63

:   585:A  27 CYS  HA  :A  38 CYS  HA  :   -0.443:       74

:   585:A  30 VAL  O   :A  30 VAL 2HG1 :   -0.435:       71
#sum2 ::8.55 clashscore : 0.00 clashscore B
```

List of bad contacts calculated by MAGE for model $num_n

```
/farm/software/bin/probe

:   585:A  13 TYR  O   :A  15 THR 3HG2 :   -0.621:       75

:   585:A  26 ILE 1HD1 :A  24 LYS 1HE  :   -0.603:       33

:   585:A   2 ALA 1HB  :A   6 GLU 2HB  :   -0.505:       61

:   585:A  27 CYS  HA  :A  38 CYS  HA  :   -0.495:       40

:   585:A  35 VAL 3HG1 :A  35 VAL  O   :   -0.495:       44
:   585:A  35 VAL  O   :A  35 VAL 2HG2 :   -0.475:       71

:   585:A   4 LYS  O   :A   5 ASN  CG  :   -0.415:       71
#sum2 ::11.97 clashscore : 3.27 clashscore B
```

List of bad contacts calculated by MAGE for model $num_n

```
/farm/software/bin/probe

:   585:A  27 CYS  HA  :A  38 CYS  HA  :   -0.569:       44

:   585:A  13 TYR  O   :A  15 THR 3HG2 :   -0.504:       64

:   585:A  26 ILE  O   :A  26 ILE 3HG2 :   -0.478:       72

:   585:A  35 VAL 2HG1 :A  35 VAL  O   :   -0.439:       64
#sum2 ::6.84 clashscore : 0.00 clashscore B
```

List of bad contacts calculated by MAGE for model $num_n

```
/farm/software/bin/probe

:   585:A  29 THR  CG2 :A  35 VAL 2HG1 :   -0.670:       73
:   585:A  29 THR 3HG2 :A  35 VAL 2HG1 :   -0.648:       73
:   585:A  35 VAL  CG1 :A  29 THR 3HG2 :   -0.494:       73
:   585:A  35 VAL  HA  :A  29 THR 3HG2 :   -0.478:       73

:   585:A   2 ALA 1HB  :A   6 GLU 2HB  :   -0.502:       52

:   585:A  31 PRO 1HD  :A  30 VAL  CG2 :   -0.431:       63

:   585:A  27 CYS  HA  :A  38 CYS  HA  :   -0.430:       72
#sum2 ::11.97 clashscore : 0.00 clashscore B
```

List of bad contacts calculated by MAGE for model $num_n

```
/farm/software/bin/probe

:   585:A  13 TYR  O   :A  15 THR 3HG2 :   -0.644:       14

:   585:A  27 CYS  HA  :A  38 CYS  HA  :   -0.544:       35

:   585:A  35 VAL 3HG1 :A  35 VAL  O   :   -0.503:       40
:   585:A  35 VAL  O   :A  35 VAL 2HG2 :   -0.454:       74

:   585:A  41 MET  CB  :A  26 ILE 2HD1 :   -0.478:       64
:   585:A  41 MET 1HB  :A  26 ILE 2HD1 :   -0.405:       64

:   585:A   4 LYS  O   :A   5 ASN  CB  :   -0.429:       22

:   585:A  30 VAL  O   :A  30 VAL 2HG1 :   -0.425:       13
#sum2 ::13.68 clashscore : 12.82 clashscore B
```

List of bad contacts calculated by MAGE for model $num_n

```
/farm/software/bin/probe

:   585:A  27 CYS  HA  :A  38 CYS  HA  :   -0.533:       62

:   585:A  26 ILE  O   :A  26 ILE 3HG2 :   -0.504:       53

:   585:A   4 LYS  O   :A   5 ASN  CB  :   -0.473:       72

:   585:A   2 ALA 3HB  :A  19 GLY  O   :   -0.450:        4

:   585:A  15 THR 1HG2 :A  36 ASN  OD1 :   -0.448:       53
#sum2 ::8.55 clashscore : 3.60 clashscore B
```

List of bad contacts calculated by MAGE for model $num_n

```
/farm/software/bin/probe

:   585:A  35 VAL  HA  :A  29 THR 3HG2 :   -0.722:       32
```

```
:   585:A  29 THR  CG2 :A  35 VAL 2HG2 :   -0.621:       24
:   585:A  35 VAL  CA  :A  29 THR 3HG2 :   -0.571:       32
:   585:A  29 THR 3HG2 :A  35 VAL 2HG2 :   -0.494:       24

:   585:A   4 LYS  O   :A   5 ASN  CB  :   -0.449:       62

:   585:A  27 CYS  HA  :A  38 CYS  HA  :   -0.424:       50
#sum2 ::10.26 clashscore : 13.42 clashscore B
```

List of bad contacts calculated by MAGE for model $num_n

```
/farm/software/bin/probe

:   585:A  27 CYS  HA  :A  38 CYS  HA  :   -0.447:       51

:   585:A  35 VAL 2HG1 :A  35 VAL  O   :   -0.445:       35

:   585:A  31 PRO 1HD  :A  30 VAL  CG2 :   -0.443:       54
#sum2 ::5.13 clashscore : 4.00 clashscore B
```

Output from PDB validation software

Summary from PDB validation

```
                                                       Dec. 26, 08:57:13 2014

[ Text modified to reflect that this was run under PSVS - Aneerban Bhattacharya: Dec 2005 ]


The following checks were made on :
-----------------------------------------

CLOSE CONTACTS

==> Distances smaller than 2.2 Angstroms are considered as close contacts
    for heavy atoms, 1.6 Angstroms for hydrogens.

      none



DISTANCES AND ANGLES

We have checked your intra and intermolecular distances and angles with the
procedures currently in place at PDB:

==> Bond and angle checks are performed by first computing the average rms
    error for all bonds and angles relative to standard values for nucleotide
    units [L. Clowney et al., Geometric Parameters in Nucleic Acids: Nitrogenous
    Bases, J.Am.Chem.Soc. 1996, 118, 509-518; A. Gelbin et al., Geometric
    Parameters in Nucleic Acids: Sugar and Phosphate Constituents, J.Am.Chem.Soc.
    1996, 118, 519-529] and amino acid units [R.A. Engh and R. Huber, Accurate
    Bond and Angle Parameters for X-ray protein structure refinement, Acta
    Crystallogr. 1991, A47, 392-400]. Any bond or angle which deviates from the
    dictionary values by more than six times this computed rms error is
    identified as an outlier.
```

*** Covalent Bond Lengths:

The RMS deviation for covalent bonds relative to the standard dictionary is 0.001 Angstroms

The following table contains a list of the covalent bonds greater than 6.0*RMSD.

Deviation	Residue Name	Chain ID	Sequence Number	Model	AT1	-	AT2	Bond Distance	Dictionary Value
-0.005	PRO	A	31	1	CG	-	CD	1.498	1.503
-0.005	PRO	A	31	2	CG	-	CD	1.498	1.503
-0.006	PRO	A	31	3	CG	-	CD	1.497	1.503
-0.005	PRO	A	31	4	CG	-	CD	1.498	1.503
-0.005	PRO	A	31	5	CG	-	CD	1.498	1.503
-0.005	PRO	A	31	6	CG	-	CD	1.498	1.503
-0.006	PRO	A	31	7	CG	-	CD	1.497	1.503
-0.005	PRO	A	31	8	CG	-	CD	1.498	1.503
-0.005	PRO	A	31	9	CG	-	CD	1.498	1.503
-0.005	PRO	A	31	10	CG	-	CD	1.498	1.503
-0.006	PRO	A	31	11	CG	-	CD	1.497	1.503
-0.006	PRO	A	31	12	CG	-	CD	1.497	1.503
-0.005	PRO	A	31	14	CG	-	CD	1.498	1.503
-0.005	PRO	A	31	15	CG	-	CD	1.498	1.503
-0.005	PRO	A	31	16	CG	-	CD	1.498	1.503
-0.005	PRO	A	31	17	CG	-	CD	1.498	1.503
-0.005	PRO	A	31	18	CG	-	CD	1.498	1.503
-0.005	PRO	A	31	20	CG	-	CD	1.498	1.503

*** Covalent Angle Values:

The RMS deviation for covalent angles relative to the standard dictionary is 0.2 degrees.

All covalent bond angles lie within a 6.0*RMSD range about the standard dictionary values.

TORSION ANGLES

The torsion angle distributions have been checked. The postscript file of the conformation rings showing the torsion angle distributions will be sent in a separate E-mail message.

CHIRALITY

The chirality has been checked. O1P, O2P, and hydrogen atoms which do not follow the convention defined in the IUBMB (Liebecq, C. Compendium of Biochemical Nomenclature and Related Documents, 2nd ed.; Portland Press: London and Chapel Hill, 1992) and IUPAC nomenclature (J.L. Markley, A. Bax, Y. Arata, C.W. Hilbers, R. Kaptein, B.D. Sykes, P.E. Wright and K. Wüthrich, Recommendations for the Presentation of NMR Structures of Proteins and Nucleic Acids, Pure & Appl. Chem., Vol. 70, pp. 117-142, 1998) have been

standardized. Any other stereochemical violations are listed below.

E/Z NOMENCLATURE

E/Z nomenclature of hydrogens and/or nitrogens on Arg, Asn or Gln residues needs to be corrected to conform with the standard for E/Z orientation presented in [J.L. Markley, et al., Recommendations for the Presentation of NMR Structures of Proteins and Nucleic Acids, Pure & Appl. Chem., 1998, 70, 117-142].

Model	Chain	Residue Name	Residue Number	Atom Name	Original Atom Name
1	A	ASN	5	1HD2	
1	A	ASN	5	2HD2	
1	A	ASN	10	1HD2	
1	A	ASN	10	2HD2	
1	A	ASN	22	1HD2	
1	A	ASN	22	2HD2	
1	A	ASN	36	1HD2	
1	A	ASN	36	2HD2	
2	A	ASN	5	1HD2	
2	A	ASN	5	2HD2	
2	A	ASN	10	1HD2	
2	A	ASN	10	2HD2	
2	A	ASN	22	1HD2	
2	A	ASN	22	2HD2	
2	A	ASN	36	1HD2	
2	A	ASN	36	2HD2	
3	A	ASN	5	1HD2	
3	A	ASN	5	2HD2	
3	A	ASN	10	1HD2	
3	A	ASN	10	2HD2	
3	A	ASN	22	1HD2	
3	A	ASN	22	2HD2	
3	A	ASN	36	1HD2	
3	A	ASN	36	2HD2	
4	A	ASN	5	1HD2	
4	A	ASN	5	2HD2	
4	A	ASN	10	1HD2	
4	A	ASN	10	2HD2	
4	A	ASN	22	1HD2	
4	A	ASN	22	2HD2	
4	A	ASN	36	1HD2	
4	A	ASN	36	2HD2	
5	A	ASN	5	1HD2	
5	A	ASN	5	2HD2	
5	A	ASN	10	1HD2	
5	A	ASN	10	2HD2	
5	A	ASN	22	1HD2	
5	A	ASN	22	2HD2	
5	A	ASN	36	1HD2	
5	A	ASN	36	2HD2	
6	A	ASN	5	1HD2	
6	A	ASN	5	2HD2	
6	A	ASN	10	1HD2	
6	A	ASN	10	2HD2	
6	A	ASN	22	1HD2	
6	A	ASN	22	2HD2	
6	A	ASN	36	1HD2	
6	A	ASN	36	2HD2	
7	A	ASN	5	1HD2	

7	A	ASN	5	2HD2
7	A	ASN	10	1HD2
7	A	ASN	10	2HD2
7	A	ASN	22	1HD2
7	A	ASN	22	2HD2
7	A	ASN	36	1HD2
7	A	ASN	36	2HD2
8	A	ASN	5	1HD2
8	A	ASN	5	2HD2
8	A	ASN	10	1HD2
8	A	ASN	10	2HD2
8	A	ASN	22	1HD2
8	A	ASN	22	2HD2
8	A	ASN	36	1HD2
8	A	ASN	36	2HD2
9	A	ASN	5	1HD2
9	A	ASN	5	2HD2
9	A	ASN	10	1HD2
9	A	ASN	10	2HD2
9	A	ASN	22	1HD2
9	A	ASN	22	2HD2
9	A	ASN	36	1HD2
9	A	ASN	36	2HD2
10	A	ASN	5	1HD2
10	A	ASN	5	2HD2
10	A	ASN	10	1HD2
10	A	ASN	10	2HD2
10	A	ASN	22	1HD2
10	A	ASN	22	2HD2
10	A	ASN	36	1HD2
10	A	ASN	36	2HD2
11	A	ASN	5	1HD2
11	A	ASN	5	2HD2
11	A	ASN	10	1HD2
11	A	ASN	10	2HD2
11	A	ASN	22	1HD2
11	A	ASN	22	2HD2
11	A	ASN	36	1HD2
11	A	ASN	36	2HD2
12	A	ASN	5	1HD2
12	A	ASN	5	2HD2
12	A	ASN	10	1HD2
12	A	ASN	10	2HD2
12	A	ASN	22	1HD2
12	A	ASN	22	2HD2
12	A	ASN	36	1HD2
12	A	ASN	36	2HD2
13	A	ASN	5	1HD2
13	A	ASN	5	2HD2
13	A	ASN	10	1HD2
13	A	ASN	10	2HD2
13	A	ASN	22	1HD2
13	A	ASN	22	2HD2
13	A	ASN	36	1HD2
13	A	ASN	36	2HD2
14	A	ASN	5	1HD2
14	A	ASN	5	2HD2
14	A	ASN	10	1HD2
14	A	ASN	10	2HD2
14	A	ASN	22	1HD2
14	A	ASN	22	2HD2
14	A	ASN	36	1HD2

14	A	ASN	36	2HD2
15	A	ASN	5	1HD2
15	A	ASN	5	2HD2
15	A	ASN	10	1HD2
15	A	ASN	10	2HD2
15	A	ASN	22	1HD2
15	A	ASN	22	2HD2
15	A	ASN	36	1HD2
15	A	ASN	36	2HD2
16	A	ASN	5	1HD2
16	A	ASN	5	2HD2
16	A	ASN	10	1HD2
16	A	ASN	10	2HD2
16	A	ASN	22	1HD2
16	A	ASN	22	2HD2
16	A	ASN	36	1HD2
16	A	ASN	36	2HD2
17	A	ASN	5	1HD2
17	A	ASN	5	2HD2
17	A	ASN	10	1HD2
17	A	ASN	10	2HD2
17	A	ASN	22	1HD2
17	A	ASN	22	2HD2
17	A	ASN	36	1HD2
17	A	ASN	36	2HD2
18	A	ASN	5	1HD2
18	A	ASN	5	2HD2
18	A	ASN	10	1HD2
18	A	ASN	10	2HD2
18	A	ASN	22	1HD2
18	A	ASN	22	2HD2
18	A	ASN	36	1HD2
18	A	ASN	36	2HD2
19	A	ASN	5	1HD2
19	A	ASN	5	2HD2
19	A	ASN	10	1HD2
19	A	ASN	10	2HD2
19	A	ASN	22	1HD2
19	A	ASN	22	2HD2
19	A	ASN	36	1HD2
19	A	ASN	36	2HD2
20	A	ASN	5	1HD2
20	A	ASN	5	2HD2
20	A	ASN	10	1HD2
20	A	ASN	10	2HD2
20	A	ASN	22	1HD2
20	A	ASN	22	2HD2
20	A	ASN	36	1HD2
20	A	ASN	36	2HD2

OTHER IMPORTANT ISSUES

==> The following residues have missing atoms:

```
RES MOD#C SEQ           ATOMS

CYS(  1 A   1)           HG
GLU(  1 A   6)          HE2
CYS(  1 A   8)           HG
CYS(  1 A  20)           HG
```

```
CYS(   1 A   21)           HG
CYS(   1 A   25)           HG
CYS(   1 A   27)           HG
CYS(   1 A   38)           HG
CYS(   1 A   40)           HG
CYS(   2 A    1)           HG
GLU(   2 A    6)          HE2
CYS(   2 A    8)           HG
CYS(   2 A   20)           HG
CYS(   2 A   21)           HG
CYS(   2 A   25)           HG
CYS(   2 A   27)           HG
CYS(   2 A   38)           HG
CYS(   2 A   40)           HG
CYS(   3 A    1)           HG
GLU(   3 A    6)          HE2
CYS(   3 A    8)           HG
CYS(   3 A   20)           HG
CYS(   3 A   21)           HG
CYS(   3 A   25)           HG
CYS(   3 A   27)           HG
CYS(   3 A   38)           HG
CYS(   3 A   40)           HG
CYS(   4 A    1)           HG
GLU(   4 A    6)          HE2
CYS(   4 A    8)           HG
CYS(   4 A   20)           HG
CYS(   4 A   21)           HG
CYS(   4 A   25)           HG
CYS(   4 A   27)           HG
CYS(   4 A   38)           HG
CYS(   4 A   40)           HG
CYS(   5 A    1)           HG
GLU(   5 A    6)          HE2
CYS(   5 A    8)           HG
CYS(   5 A   20)           HG
CYS(   5 A   21)           HG
CYS(   5 A   25)           HG
CYS(   5 A   27)           HG
CYS(   5 A   38)           HG
CYS(   5 A   40)           HG
CYS(   6 A    1)           HG
GLU(   6 A    6)          HE2
CYS(   6 A    8)           HG
CYS(   6 A   20)           HG
CYS(   6 A   21)           HG
CYS(   6 A   25)           HG
CYS(   6 A   27)           HG
CYS(   6 A   38)           HG
CYS(   6 A   40)           HG
CYS(   7 A    1)           HG
GLU(   7 A    6)          HE2
CYS(   7 A    8)           HG
CYS(   7 A   20)           HG
CYS(   7 A   21)           HG
CYS(   7 A   25)           HG
CYS(   7 A   27)           HG
CYS(   7 A   38)           HG
CYS(   7 A   40)           HG
CYS(   8 A    1)           HG
GLU(   8 A    6)          HE2
CYS(   8 A    8)           HG
```

```
CYS(  8 A  20)        HG
CYS(  8 A  21)        HG
CYS(  8 A  25)        HG
CYS(  8 A  27)        HG
CYS(  8 A  38)        HG
CYS(  8 A  40)        HG
CYS(  9 A   1)        HG
GLU(  9 A   6)       HE2
CYS(  9 A   8)        HG
CYS(  9 A  20)        HG
CYS(  9 A  21)        HG
CYS(  9 A  25)        HG
CYS(  9 A  27)        HG
CYS(  9 A  38)        HG
CYS(  9 A  40)        HG
CYS( 10 A   1)        HG
GLU( 10 A   6)       HE2
CYS( 10 A   8)        HG
CYS( 10 A  20)        HG
CYS( 10 A  21)        HG
CYS( 10 A  25)        HG
CYS( 10 A  27)        HG
CYS( 10 A  38)        HG
CYS( 10 A  40)        HG
CYS( 11 A   1)        HG
GLU( 11 A   6)       HE2
CYS( 11 A   8)        HG
CYS( 11 A  20)        HG
CYS( 11 A  21)        HG
CYS( 11 A  25)        HG
CYS( 11 A  27)        HG
CYS( 11 A  38)        HG
CYS( 11 A  40)        HG
CYS( 12 A   1)        HG
GLU( 12 A   6)       HE2
CYS( 12 A   8)        HG
CYS( 12 A  20)        HG
CYS( 12 A  21)        HG
CYS( 12 A  25)        HG
CYS( 12 A  27)        HG
CYS( 12 A  38)        HG
CYS( 12 A  40)        HG
CYS( 13 A   1)        HG
GLU( 13 A   6)       HE2
CYS( 13 A   8)        HG
CYS( 13 A  20)        HG
CYS( 13 A  21)        HG
CYS( 13 A  25)        HG
CYS( 13 A  27)        HG
CYS( 13 A  38)        HG
CYS( 13 A  40)        HG
CYS( 14 A   1)        HG
GLU( 14 A   6)       HE2
CYS( 14 A   8)        HG
CYS( 14 A  20)        HG
CYS( 14 A  21)        HG
CYS( 14 A  25)        HG
CYS( 14 A  27)        HG
CYS( 14 A  38)        HG
CYS( 14 A  40)        HG
CYS( 15 A   1)        HG
GLU( 15 A   6)       HE2
```

```
     CYS( 15 A   8)          HG
     CYS( 15 A  20)          HG
     CYS( 15 A  21)          HG
     CYS( 15 A  25)          HG
     CYS( 15 A  27)          HG
     CYS( 15 A  38)          HG
     CYS( 15 A  40)          HG
     CYS( 16 A   1)          HG
     GLU( 16 A   6)         HE2
     CYS( 16 A   8)          HG
     CYS( 16 A  20)          HG
     CYS( 16 A  21)          HG
     CYS( 16 A  25)          HG
     CYS( 16 A  27)          HG
     CYS( 16 A  38)          HG
     CYS( 16 A  40)          HG
     CYS( 17 A   1)          HG
     GLU( 17 A   6)         HE2
     CYS( 17 A   8)          HG
     CYS( 17 A  20)          HG
     CYS( 17 A  21)          HG
     CYS( 17 A  25)          HG
     CYS( 17 A  27)          HG
     CYS( 17 A  38)          HG
     CYS( 17 A  40)          HG
     CYS( 18 A   1)          HG
     GLU( 18 A   6)         HE2
     CYS( 18 A   8)          HG
     CYS( 18 A  20)          HG
     CYS( 18 A  21)          HG
     CYS( 18 A  25)          HG
     CYS( 18 A  27)          HG
     CYS( 18 A  38)          HG
     CYS( 18 A  40)          HG
     CYS( 19 A   1)          HG
     GLU( 19 A   6)         HE2
     CYS( 19 A   8)          HG
     CYS( 19 A  20)          HG
     CYS( 19 A  21)          HG
     CYS( 19 A  25)          HG
     CYS( 19 A  27)          HG
     CYS( 19 A  38)          HG
     CYS( 19 A  40)          HG
     CYS( 20 A   1)          HG
     GLU( 20 A   6)         HE2
     CYS( 20 A   8)          HG
     CYS( 20 A  20)          HG
     CYS( 20 A  21)          HG
     CYS( 20 A  25)          HG
     CYS( 20 A  27)          HG
     CYS( 20 A  38)          HG
     CYS( 20 A  40)          HG
==> The following residues have extra atoms:

     RES MOD#C SEQ          ATOMS

     CYS(  1 A   1)           H
     CYS(  2 A   1)           H
     CYS(  3 A   1)           H
     CYS(  4 A   1)           H
     CYS(  5 A   1)           H
     CYS(  6 A   1)           H
```

```
    CYS(  7 A   1)          H
    CYS(  8 A   1)          H
    CYS(  9 A   1)          H
    CYS( 10 A   1)          H
    CYS( 11 A   1)          H
    CYS( 12 A   1)          H
    CYS( 13 A   1)          H
    CYS( 14 A   1)          H
    CYS( 15 A   1)          H
    CYS( 16 A   1)          H
    CYS( 17 A   1)          H
    CYS( 18 A   1)          H
    CYS( 19 A   1)          H
    CYS( 20 A   1)          H


TboIT1.pdb: Missing KEYWDS records

TboIT1.pdb: Missing TITLE record
```
